# Yield and Coverage of Active Case Finding Interventions for Tuberculosis Control:A Systematic Review and Meta-analysis

**DOI:** 10.1155/2022/9947068

**Published:** 2022-06-30

**Authors:** Ruth W. Deya, Linnet N. Masese, Walter Jaoko, Jeremiah Chakaya Muhwa, Lilian Mbugua, David J. Horne, Susan M. Graham

**Affiliations:** ^1^University of Nairobi, Institute of Tropical and Infectious Diseases, Nairobi, Kenya; ^2^University of Washington, Department of Medicine, Seattle, USA; ^3^University of Nairobi, Department of Medical Microbiology, Nairobi, Kenya; ^4^Kenyatta University, Medicine, Dermatology, Psychiatry and Therapeutics, Nairobi, Kenya; ^5^Liverpool School of Tropical Medicine, Liverpool, UK; ^6^University of Washington, Department of Global Health, Seattle, WA, USA; ^7^University of Washington, Department of Epidemiology, Seattle, WA, USA

## Abstract

**Background:**

Active case finding (ACF) for tuberculosis (TB) is a key strategy to reduce diagnostic delays, expedite treatment, and prevent transmission.

**Objective:**

Our objective was to identify the populations, settings, screening and diagnostic approaches that optimize coverage (proportion of those targeted who were screened) and yield (proportion of those screened who had active TB) in ACF programs.

**Methods:**

We performed a comprehensive search to identify studies published from 1980-2016 that reported the coverage and yield of different ACF approaches. For each outcome, we conducted meta-analyses of single proportions to produce estimates across studies, followed by meta-regression to identify predictors. *Findings.* Of 3,972 publications identified, 224 met criteria after full-text review. Most individuals who were targeted successfully completed screening, for a pooled coverage estimate of 93.5%. The pooled yield of active TB across studies was 3.2%. Settings with the highest yield were internally-displaced persons camps (15.6%) and healthcare facilities (6.9%). When compared to symptom screening as the reference standard, studies that screened individuals regardless of symptoms using microscopy, culture, or GeneXpert®MTB/RIF (Xpert) had 3.7% higher case yield. In particular, microbiological screening (usually microscopy) as the initial test, followed by culture or Xpert for diagnosis had 3.6% higher yield than symptom screening followed by microscopy for diagnosis. In a model adjusted for use of Xpert testing, approaches targeting persons living with HIV (PLWH) had a 4.9% higher yield than those targeting the general population. In all models, studies targeting children had higher yield (4.8%-5.7%) than those targeting adults.

**Conclusion:**

ACF activities can be implemented successfully in various populations and settings. Screening yield was highest in internally-displaced person and healthcare settings, and among PLWH and children. In high-prevalence settings, ACF approaches that screen individuals with laboratory tests regardless of symptoms have higher yield than approaches focused on symptomatic individuals.

## 1. Introduction

In 2019, the World Health Organization (WHO) estimated global tuberculosis (TB) incidence at 10.0 million cases [1]. Although TB incidence declined globally by approximately 2.3% between 2018 and 2019, the goal of the WHO END TB Strategy is to reduce incidence by 10% per year by 2025. TB transmission is facilitated by the late diagnosis of many active TB cases [2–4]. Moreover, contacts of index cases with early-stage active TB are often missed due to low sensitivity of sputum microbiological tests [5]. Although efforts to increase detection have recently accelerated with the “Find. Treat. All” Initiative [6], TB case reporting remains suboptimal: only 7.1 million (71%) of new TB cases were reported to the WHO in 2019 [1, 6–8].

If left untreated, a person with infectious TB will transmit *Mycobacterium tuberculosis* to 10-15 people per year [1]. To decrease transmission, active case finding (ACF) strategies may be required. ACF is defined as any strategy to search for undiagnosed active TB disease in a defined population, usually high-risk groups, high-prevalence congregate settings or communities, or contacts of TB patients [9–11]. After cases of active TB diseases have been diagnosed, ACF activities may be followed by testing for and treatment of latent TB infection, as is often done during contact investigations [12]. Unlike countries with low TB incidence, most low- and middle-income countries do not conduct contact investigations and have placed a low priority on ACF activities [12, 13].

In 2018, the WHO issued a recommendation that high-burden countries evaluate household contacts of bacteriologically confirmed pulmonary TB cases aged 5 years and over, treating them for active or latent TB infection as indicated [6]. Planning additional ACF activities will require evaluation of TB prevalence in specific settings and populations, consideration of optimal diagnostic tests, and assessment of the cost-effectiveness of such activities [8, 14].

While studies show that screening increases case-finding in the short-term and identifies less severe cases [15, 16], there is no published meta-analysis of factors associated with higher yield (i.e., proportion of those screened who had active TB) and coverage (i.e., proportion of those targeted who were screened) of ACF activities. An analysis summarizing evidence from the published literature on the yield and coverage of ACF activities in countries with high TB prevalence would inform and help focus the efforts of policymakers and TB program managers faced with the dual TB-HIV epidemic to END TB. Our objective in the current study was therefore to identify the populations, settings, and screening and diagnostic approaches that optimize yield and coverage in ACF programs.

## 2. Methods

### 2.1. Inclusion criteria

This systematic review followed PRISMA guidelines [17]. Studies were eligible for inclusion if they were conducted in a country with high TB prevalence (>100 per 100,000) and reported results of an intervention designed to detect undiagnosed active TB. Following the WHO definition of systematic screening for active TB, we defined ACF as “the systematic identification of people at risk for TB disease, in a predetermined target group, by assessing symptoms and using tests, examinations or other procedures that can be applied rapidly” [18]. Interventions could be any TB screening method or combination of methods, including symptom screening, clinical assessment, chest X-ray (CXR), sputum acid-fast bacillus (AFB) microscopy, sputum culture, or GeneXpert® MTB/RIF testing (Xpert), followed by diagnostic work-up and treatment if indicated. We included studies reporting at least one of the following primary outcomes: (1) number of newly diagnosed active TB cases, (2) screening yield, or (3) screening coverage. Populations targeted for screening were categorized as: general population, contacts of active TB cases, individuals at high risk for active TB (e.g., persons living with HIV [PLWH], persons with diabetes mellitus, pregnant women, hospital inpatients), individuals at high risk for TB exposure (e.g., refugees, homeless persons, prisoners, residents of other congregate settings), or workers in high-risk settings for TB exposure (e.g., miners, healthcare workers). Population-based surveys, observational studies (i.e., cross-sectional or cohort studies) and randomized trials were eligible. We excluded studies whose primary outcome was the diagnosis of latent TB infection including those only testing for TB infection (with a TB skin test or interferon-gamma release assay).

### 2.2. Search strategy

With the help of a research librarian, we searched PubMed, EMBASE, Global Health Database, Cochrane Library Central Register of Controlled Trials, Scopus and the WHO Library, using MeSH terms for PubMed and comparable terms for the other databases. We consulted the WHO International Clinical Trials Registry Platform for ongoing trials. Full details of the search strategy are included in Supplemental Material 1. We limited the search to English-language studies conducted between January 1, 1980 (after the emergence of the HIV epidemic) and September 18, 2016. For studies that used different screening modalities for different populations, including separate arms of clinical trials, results were evaluated separately for each population. If two different papers reported data from the same study population, we included only the latter one or the one with the most complete outcome reporting.

### 2.3. Citation screening and data abstraction

We used EndNote to import citations and remove duplicates. Two researchers screened each abstract for relevance to the study of ACF outcomes, with a third reviewer resolving discrepancies. If the results of abstract screening were unclear, the article was included for the next stage of screening. After identification of potentially eligible studies, four reviewers independently conducted full text review on 40 randomly selected papers to standardize methods for confirming eligibility, extracting data, and rating study quality. We extracted study description, setting, and study populations included from included papers. If a study conducted screening activities in two distinct populations (e.g., children and adults), these were considered two different “populations” with separate outcomes. For each population, we abstracted the tests, examinations or other procedures that were applied, and the basis upon which individuals were selected for each test, examination, or procedures were applied. The screening method was defined as any test applied to the entire study population to identify individuals who would receive further active TB workup. The diagnostic method was defined as any TB diagnostic test applied to individuals who screened positive with the initial screening method. We also abstracted the number targeted for TB screening; number completing TB screening; number of new active TB cases identified; number initiating and completing TB treatment (if reported); number of cases reported as sputum-, culture- or Xpert-positive; number with CXR abnormalities; and data on rifampicin resistance or multi-drug-resistant TB (MDR-TB) (i.e., resistance to at least rifampicin and isoniazid).

### 2.4. Quality review

A modified Joanna Briggs Institute Critical Appraisal Checklist for Studies Reporting Prevalence Data was used to rate study quality [19]. Studies were rated as “yes,” “no,” or “unclear” on the following criteria: adequate description of subjects and setting, use of valid TB screening and diagnostic methods, use of standardized and reliable screening and diagnosis measures, and adequacy of screening participation (defined as coverage ≥80%). A final overall quality rating was assigned to each included article: “high” if all four quality metrics were rated as “yes”, “moderate” if any metric was rated “unclear”, and “low” if any metric was rated “no”.

### 2.5. Screening and diagnostic categories

Based on the abstracted chart data, we assigned one screening method and one diagnostic method category to each population screened. We divided screening modalities into four categories: (1) symptom screening, (2) microbiological testing (microscopy, culture, or Xpert) with or without symptoms screening, (3) CXR with or without symptoms screening, and (4) tuberculin skin tests (TST). Any study that used a microbiological test **(**microscopy, culture, or GeneXpert MTB/RIF) was assigned as “lab testing”; any study that used CXR and not lab tests was assigned as “CXR”. Any study that used symptom screening and not lab tests or CXR was assigned as “symptom screening”. Finally, one remaining study was assigned as “TST” because it did not use lab testing, CXR or symptom screening to identify individuals who qualify for next step in TB diagnosis.

**Figure 1:**
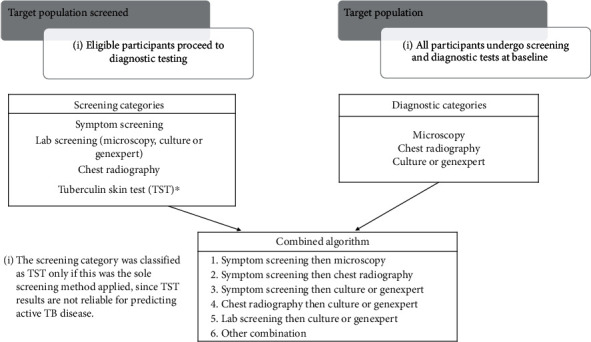
Combined Screening and Diagnostic Algorithm Variable.

We divided diagnostic modalities into three categories: (1) culture and/or Xpert, (2) microscopy and (3) CXR. Any study that used culture and/or Xpert was assigned as “culture and/or Xpert”; any study that used microscopy and neither culture nor Xpert was assigned as “microscopy” and any study that used CXR and not culture, Xpert or microscopy was assigned as “CXR”. Finally, we created a combined “algorithm” variable incorporating both screening and diagnostic modalities in the six categories, as illustrated in Figure 1.

### 2.6. Statistical analysis

Descriptive statistics were used to summarize data, with number and percentage for categorical data and median and interquartile range for continuous data. Study attributes summarized included study design; WHO geographical region; setting (community, hospital or clinic, prison or residential facility, workplace, other settings); type of population targeted (contacts, PLWH, general population, high risk for TB exposure, high risk for active TB); and participant age (adults [defined as age ≥15 years], children or both) study quality and year of publication.

We conducted a separate meta-analysis of single proportions for each of the two outcomes (yield and coverage). For each specific population, the two outcomes of interest were expressed as proportions, using the appropriate numerators and denominators abstracted from the publication. After excluding studies with missing numerators or denominators, we used random effects modeling to derive pooled yield and pooled coverage proportion estimates across populations. This was done using Stata's ‘metaprop' command to calculate the pooled estimate after Freeman-Tukey Double Arcsine Transformation to stabilize the variances [20]. Score-based confidence intervals [21] were calculated based on a random effects model using the method of DerSimonian and Laird, with the estimate of heterogeneity being taken from the inverse-variance fixed-effect model [22]. Heterogeneity across populations was evaluated using Cochrane's Q test statistic and the I^2^ statistic.

Meta-analysis was followed by meta-regression using the study and population attributes described above (i.e., study design, WHO region, recruitment setting, type of population screened, population age category, year of publication, study quality and either screening and diagnostic modality as two separate variables or as a combined algorithm variable) as independent predictors of yield and coverage. We used the ‘metareg' function in Stata to perform a random-effects meta-regression using aggregate-level data while taking into account the within-study standard error of the dependent variable (i.e., yield or coverage) [23]. Results reported as beta coefficients with 95% confidence intervals and interpretable as differences relative to the reference category. Predictors of yield and of coverage that were significant at p <0.10 in bivariable analysis were included in multivariable analysis. We ran three multivariable models. Model 1 included screening and diagnostic modalities as separate predictors. Model 2 used the combined algorithm variable that incorporated both screening and diagnostic modality. Model 3 evaluated the use of Xpert for diagnosis as a binary yes/no predictor, to determine if Xpert was an important driver of increased yield. Two sensitivity analyses were conducted, one excluding studies of populations with children (since diagnostic approaches in children differ from those in adults) and one excluding low-quality studies. Statistical analyses were performed using Stata version 16.0 (College Station, Texas).

## 3. Results

### 3.1. Included studies

**Figure 2:**
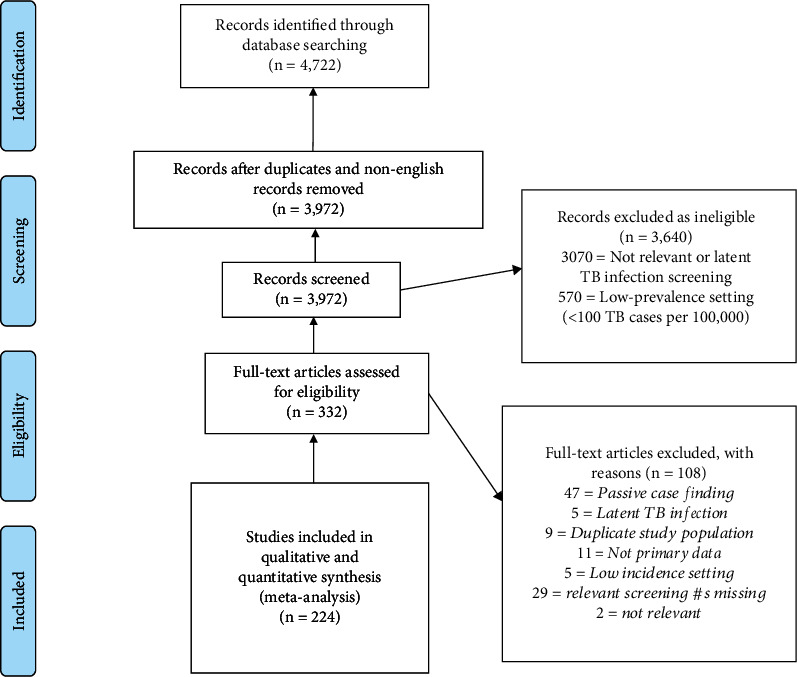
PRISMA Diagram.

Our search identified 4,722 records, of which 750 were duplicates or not in English language. We screened the remaining 3,972 abstracts and identified 224 papers for inclusion in analysis (Figure 2). These 224 studies reported coverage results for 256 unique populations and yield for 277 unique populations. As an example of a study that presented results of different populations, Banu et al. [24] screened inmates at the time of prison entry and also conducted active case finding among inmates already incarcerated, and these were counted as two separate populations. Overall, 25,652,139 individuals were screened, with 55,549 new TB cases identified.  Table 1(a) presents a summary of study attributes and Table 1(b) presents a summary of populations included in the studies. Supplemental Material 2 provides a detailed list of all included studies.

Most studies took place in Africa (68.3%) or Southeast Asia (22.7%) and were observational cross-sectional or cohort study designs (80.0%). Sites were the community (52.7%), hospitals or primary health centers (32.9%), prisons or other residential institutions (7.6%), workplaces (6.1%), or camps for internally-displaced persons (0.7%). No studies focused on immigrants or refugee settings. Adults comprised 180 populations (65.0%), both adults and children 56 populations (20.2%), and children 41 populations (14.8%).

Most studies used one of six screening and diagnostic algorithms, with the two most commonly used algorithms being [a] symptom screening followed by sputum culture or Xpert (30.0%) and [b] symptom screening followed by sputum microscopy (19.5%). Only one study, by Nachega et al., applied TST as the first screening step, using clinical criteria, CXR, microscopy and culture to confirm active TB status among individuals who were TST- positive [25]. HIV status was assessed in 141 populations (50.9%). Few studies reported treatment initiation (88 or 31.8%) or treatment completion rates (27 or 9.8%). Most of the 224 studies were of high quality (136 or 60.7%), while 25 were of moderate quality (11.2%) and 63 were of low quality (28.1%).

### 3.2. Pooled results for ACF coverage

**Figure 3:**
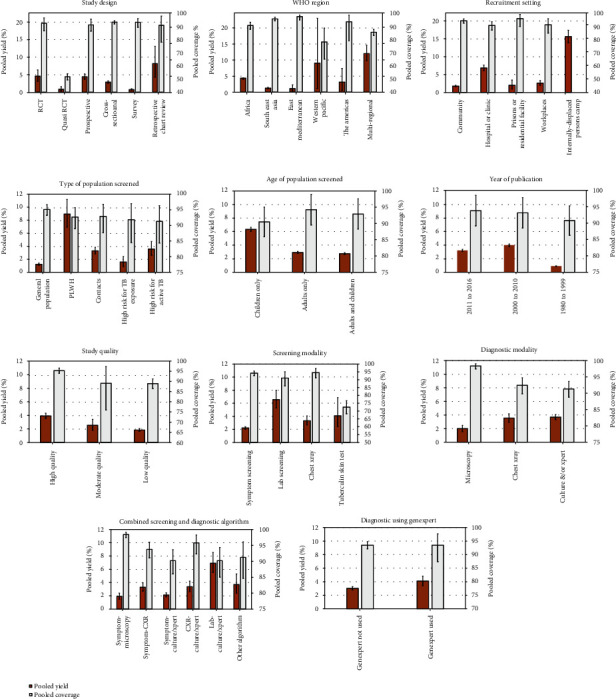
Pooled Estimates of Yield and Coverage by Study and Population Attributes; Coverage is defined as total number of people of screened/total number of people targeted for screening. Yield is defined as total number of people with active TB/total number of people screened. Bars indicate 95% confidence intervals of random effects meta-analyses.

Figure 3 presents pooled coverage estimates (white bars) by region and other population and study attributes. Overall, the pooled coverage among the 256 populations that reported coverage was 93.5% (95% confidence interval [CI], 92.2% – 94.7%). Coverage was relatively high (>90%) in most studies, but was notably lower in the one study that used a quasi-randomized controlled trial (RCT) design (52.0%) and reported coverage data. This study, called the ZAMSTAR study, tested a community-level enhanced TB case-finding approach including sputum testing versus household-level combined TB and HIV care in Zambia and South Africa, and had a calculated coverage of 52.0% [26]. Supplemental material 3 includes details of the numbers targeted and screened in each category.

### 3.3. Meta-regression of ACF coverage

Supplemental material 4 includes details of the meta-regression of ACF coverage. Predictors of coverage significant in bivariable analyses included study type (p =0.002), study quality (0.003) diagnostic modality (p =0.02), and algorithm category (p =0.02). In a multivariable model including only study type, diagnostic modality and quality as predictors, coverage in quasi-RCT studies was lower by 38.6% compared to cross-sectional studies. Studies that used culture or Xpert for diagnosis had 9.7% lower coverage than studies using microscopy for diagnosis. Moderate and low-quality studies had 12.1% and 10.3% lower coverage than high quality studies, respectively, with a similar trend in all 3 models. In a model including study type, algorithm category and quality as predictors, coverage was lower by 37.8% in the quasi-RCT studies compared to cross-sectional studies. Coverage was significantly lower by 12.1% when symptom screening was followed by culture or Xpert compared to symptom screening followed by microscopy and lower by 11.4% when laboratory testing was the initial screening method.

### 3.4. Pooled results for ACF yield

Figure 3 presents pooled yield estimates (dark orange bars) by region and other population and study attributes. The pooled yield of new TB cases in all 277 populations was 3.2% (95% CI, 2.9%–3.4%). The highest yield was in internally-displaced persons settings (15.6%) and in a multiregional study, at 12.0% [27], while the lowest yield was in the six populations included in the two quasi-RCT studies and in studies published from 1980-1999 (both 0.9%). The screening approach with the lowest yield was symptom screening followed by microscopy (1.9%). In terms of settings, the highest yield was reported in ACF activities at internally-displaced persons camp (15.6%) and healthcare facilities (6.9%). Active case finding among PLWH had the highest yield (9.0%) in terms of population screened. Heterogeneity was high (overall *I^2^* = 99.7%) due to the wide variety of populations, settings, screening and diagnostic approaches, and study designs. Yield was lower in studies rated as low quality, but study quality as a categorical variable was not a significant predictor of yield in regression analyses. Supplemental material 5 includes details of the numbers screened and diagnosed in each category.

### 3.5. Meta-regression of ACF yield

Table 2 presents bivariable and multivariable meta-regression results for the pooled yield outcome. Predictors of yield that were significant in bivariable analysis were study design (p =0.08), WHO geographical region (p =0.02), recruitment setting (p <0.001), population screened (p <0.001), age (p =0.02), screening modality (p =0.01), and algorithm used (p =0.02). The three multivariable models are presented in separate columns of Table 2. In Model 1, which included screening and diagnostic modalities as separate predictors, studies that screened children exclusively had 5.7% higher yield when compared with studies that screened only adults and studies that screened individuals regardless of symptoms using microscopy, culture, or Xpert had 3.7% higher case yield than studies that focused on symptoms as the initial screen. In Model 2, which used the combined algorithm variable incorporating both screening and diagnostic modality, studies that screened children exclusively had 5.6% higher yield when compared with studies that screened only adults, and studies that used microbiological screening (94% of which used microscopy) as the initial test, followed by culture or Xpert for diagnosis had 3.6% higher yield than symptom screening followed by microscopy for diagnosis. In Model 3, where the binary Xpert variable indicating diagnosis by Xpert was used rather than the screening or approach variables, studies that screened children exclusively had 4.8% higher yield when compared with studies that screened only adults, and approaches targeting PLWH had a 4.9% higher yield than those targeting the general population. Of note, age was the only predictor of yield significant in all multivariable models (at p =0.005, p =0.005 and p =0.02 in multivariable Models 1, 2 and 3, respectively). Sensitivity analyses excluding studies of children and excluding low-quality studies had similar results to the main analyses (details not shown).

## 4. Discussion

Our systematic review and meta-analysis identified the pooled yield and coverage of various ACF approaches in 277 study populations across 224 studies carried out in 30 high-incidence countries. We highlight how various screening and diagnostic modalities perform when employed in a variety of settings, with respect to both coverage and yield. ACF coverage was generally high, with a pooled estimate of 93.5%, indicating high acceptability in most studies. ACF yield was estimated at 3.2% overall, and was higher in studies targeting children than in those targeting adults; this finding was consistent in all meta-regression analyses. ACF yield was also significantly higher in PLWH compared to the general population, and yield estimates were high in health care settings and settings where internally-displaced persons were temporarily settled. In addition, ACF yield was higher when microbiological screening methods were applied to individuals regardless of symptoms. These findings should mitigate concerns about the higher cost of sensitive and specific laboratory tests, especially when ACF targets populations or settings where TB transmission or incidence rates are high.

Coverage, or successful screening completion, is influenced by many factors, including the acceptability of screening and complexity of screening procedures. In the studies we reviewed, ACF coverage was generally high, reflecting high acceptability of screening in high TB burden countries. While coverage estimates were somewhat lower in studies that screened using laboratory-based methods, this was likely due to barriers to access (e.g., transportation costs, limited business hours) for repeat sample collections [28]. Although microscopic detection of AFB is more specific for TB diagnosis than the presence of TB symptoms, laboratory-based screening can introduce barriers to screening completion, compromising coverage and early TB detection [28–30]. In our review, recruitment settings with the highest coverage were prisons and residential facilities (96.4%), supporting ACF activities in these settings. Studies in our review with low coverage cited challenges with logistics, specimen handling, and reaching highly mobile populations. Specifically, the ZAMSTAR Quasi RCT was an ambitious study that enumerated and sought to consent individuals in 24 communities in Zambia and South Africa. In this study using community enhanced case finding, household-based screening and clinic-based interventions, screening completion was impacted by refusals to participate, long distances for transporting specimen, missing or contaminated sputum culture samples excluded for failure to meet quality assurance standards [26]. Successful screening completion requires simple methods, including point-of care tests that are easy to use in the field.

Yield, or the successful identification of new TB cases, is the more important of our two outcomes, especially given the high coverage rates in most studies. Interestingly, while most studies in this systematic review followed the basic WHO algorithm (i.e., symptom screening followed by microscopy), this approach led to the lowest pooled yield of new TB cases (1.9%). We found that the use of microbiological tests for screening was associated with higher yield, with the highest yield of new TB cases found in studies that used microbiological methods for both screening and diagnosis (6.9%). In the 40 (14.4%) studies that used Xpert as a diagnostic method, yield was estimated at 4.1%, compared to 3.0% in all studies that did not use Xpert. Our results suggest that incorporating laboratory-based testing into ACF screening activities would be a worthwhile investment for most high-burden countries, given the challenge of relying on symptoms to target ACF activities.

While community-based ACF may result in low yield due to opportunistic testing by the “worried well” [31], our results demonstrate that targeting ACF to higher risk populations increases yield. For example, our study reinforces the importance of ACF activities among PLWH, which led to almost three-fold higher yield (9.0%) than the pooled average (3.2%). Regular screening for TB among PLWH should be a focus of programs where HIV co-infection is prevalent [5]. Similarly, ACF yield among children (6.3%) was double that of the averaged pooled yield for all studies in our review. One explanation may be that ACF among children was more exhaustive, often using multiple modalities; clinical, microbiological and radiological criteria in both screening and diagnostic algorithms [32]. Among the 41 studies focused on ACF among children, pooled coverage (90.5%) was high, despite complex diagnostic protocols. The high estimated yield should be interpreted in the context of two outliers which likely drove these findings: the LaCourse study of severely malnourished children (73.0%) [33] and the Topley study of pediatric contacts to adults with active TB (63.8%) [34]. A focus on high-risk children and high-risk adults should be core to ACF activities, and will likely increase their cost-effectiveness.

The TB care cascade involves not only screening and diagnosis, but also treatment initiation, completion and cure [35, 36]. Since the goal of TB screening is treatment and cure, and ultimately the interruption of transmission, we highlight gaps in reporting on the ACF cascade, with only 31.8% of studies reporting treatment initiation and even fewer (9.8%) reporting treatment completion rates. Important inferences on refining the TB care cascade and ACF logistics could be made if future ACF publications clearly reported these important parameters. Surveillance for TB drug resistance, which was reported in only 10 studies in this review, should also be incorporated into future ACF research and programs, especially in settings with a high burden of MDR-TB and extensively drug resistant TB (XDR-TB).

Our study had several limitations. First, heterogeneity was high due to the large variety of screening and diagnostic modalities, populations, settings, and study designs in our sample. We addressed this by conducting a random effects meta-analysis and meta-regression, as well as by adjusting for variables including year of publication, WHO region and study design. Second, we present only studies that were peer-reviewed and published in English. Third, 28% were rated as low quality and 8% of studies were missing data on coverage, one of our main outcomes. Fourth, the majority of published studies followed WHO recommendations on TB screening (i.e., interview for TB symptoms and HIV status) and diagnosis (i.e., microscopy or sputum culture if available) [37], and the number of studies researching ACF innovations was small during the time period of our search. Fifth, given the use of ACF and the heterogeneity in study design, population, and durations we are cannot directly compare TB yield in the research study populations to published WHO country data on annual TB case rates. We did not identify any studies of refugees or immigrants currently living in high-prevalence countries. We used a hierarchical classification of screening and diagnostic modalities making it challenging to disentangle the effects of individual modalities. However, these modalities were applied jointly and represent pragmatic TB screening or diagnostic activities.

In conclusion, ACF is an important adjunct to passive TB case detection that will be needed to improve TB control efforts globally. Approaches using microbiological testing, especially innovations such as Xpert testing, and targeting settings or populations with higher risk may increase the yield of ACF activities. Screening completion was successful in most populations and settings. In addition, the relation between ACF approaches and the full TB care cascade is an important gap in the evidence base that should be addressed in future research.

## Figures and Tables

**(a):** 

Characteristic	N (%)
Study design	
Cross-sectional	122 (54.5)
Prospective	57 (25.5)
Population – Based survey	19 (8.5)
RCT	13 (5.8)
Retrospective chart reviews	11 (4.9)
Quasi RCT	2 (0.9)
WHO region	
Africa	153 (68.3)
South East Asia	51 (22.7)
Eastern Mediterranean	10 (4.5)
Western Pacific	5 (2.2)
The Americas	4 (1.8)
Other (multi-regional study^a^)	1 (0.5)
Quality rating	
High	136 (60.7)
Moderate	25 (11.2)
Low	63 (28.1)
Year of publication	
1980 to 1999	22 (7.9)
2000 to 2010	66 (23.8)
2011 to 2016	189 (68.2)

^a^Botswana, Malawi, South Africa, Zimbabwe, India, Peru and Brazil.

**(b):** 

Characteristics of 277 populations	N (%)
Recruitment setting, by population	
Community based	146 (52.7)
Hospitals or primary health centers	91 (32.9)
Prisons and other residential institutions	21 (7.6)
Workplaces	17 (6.1)
Internally-displaced persons camps	2 (0.7)
Type of population screened	
General population	90 (32.5)
Contacts	68 (24.6)
PLWH	59 (21.3)
High risk for TB exposure^a^	31 (11.2)
High risk for active TB^b^	29 (10.5)
Population age group	
Adults only	180 (65.0)
Adults and children	56 (20.2)
Children only	41 (14.8)
HIV assessment	
Testing	132 (47.7)
Self-report	7 (2.5)
Other (chart review)	2 (0.7)
Not reported	136 (49.1)
Treatment initiation	
Reported	88 (31.8)
Not reported	189 (68.2)
Treatment completion	
Reported	27 (9.8)
Not reported	250 (90.3)
Screening modality^c^	
Symptom screening	182 (65.7)
Lab testing (microscopy, culture, or GeneXpert MTB/RIF)	58 (21.0)
CXR	36 (13.0)
TST	1 (0.3)
Diagnostic modality	
Culture &/or GeneXpert MTB/RIF	165 (59.6)
Microscopy	61 (22.0)
CXR	51 (18.4)
Screening and diagnostic algorithm	
Symptom screen – Microscopy	54 (19.5)
Symptom screen – CXR	44 (15.9)
Symptom screen – Culture/Xpert	84 (30.3)
CXR – Culture/Xpert	29 (10.5)
Lab screen^d^ – Culture/Xpert	51 (18.4)
Other^e^	15 (5.4)

CXR = Chest x-ray, RCT = Randomized control trial, TST = Tuberculin skin test ^a^ High risk for TB exposure: health care worker, prisoner, refugee. ^b^ High risk for active TB: diabetes mellitus, pregnancy, miners. ^c^ Tuberculin skin tests were included as part of screening in 5 studies classified as “lab testing,” 14 studies classified as “CXR” and 15 studies classified as “symptom screening.” ^d^ Includes initial TB screening by AFB smear (94%), culture (71%), or GeneXpert MTB/RIF (8%). ^e^ Other = microscopy for screening and diagnosis (5), CXR for screening and diagnosis (5), CXR for screening then microscopy for diagnosis (2), TST for screening then culture for diagnosis (1), and microscopy for screening then CXR for diagnosis (2).

**Table 2:** Meta-regression of Predictors of Yield of Active TB Cases following Active Case Finding Activities.

Predictors of yield proportion	Bivariable Anaylsis		Multivariable model 1: Screening and diagnosis separate		Multivariable model 2: Combined approach		Multivariable model 3: Diagnosis by GeneXpert MTB/RIF	
Overall	Beta (95% CI)	P value	Beta (95% CI)	P value	Beta (95% CI)	P value	Beta (95% CI)	P value
Study design		**0.08**		0.42		0.36		0.67
Cross-sectional	Reference		Reference		Reference		Reference	
Prospective	0.98 (-1.56 to 3.52)		-1.12 (-3.66 to 1.41)		-1.11 (-3.65 to 1.42)		-1.10 (-3.62 to 1.42)	
RCT	0.96 (-2.82 to 4.74)		0.79 (-3.14 to 4.72)		1.00 (-2.92 to 4.93)		0.02 (-3.79 to 3.83)	
Surveys	-3.55 (-7.36 to 0.27)		-2.66 (-6.43 to 1.10)		-2.51 (-6.26 to 1.24)		-2.04 (-5.73 to 1.64)	
Retrospective chart review	4.74 (-0.46 to 9.94)		0.57 (-4.70 to 5.84)		1.52 (-3.81 to 6.85)		0.84 (-4.40 to 6.08)	
Quasi RCT	-3.78 (-10.87 to 2.72)		-5.16 (-11.47 to 1.16)		-5.44 (-11.75 to 0.86)		-3.84 (-9.95 to 2.28)	
WHO region		**0.02**		0.21		0.28		0.15
Africa	Reference		Reference		Reference		Reference	
Americas	-1.2 (-7.67 to 5.26)		-3.37 (-10.52 to 3.77)		-3.18 (-10.37 to 4.00)		-2.29 (-9.44 to 4.86)	
Eastern Mediterranean	-4.31 (-9.89 to 1.28)		-3.51 (-9.03 to 2.01)		-3.37 (-8.86 to 2.12)		-3.38 (-8.88 to 2.13)	
S.E. Asia	-3.50 (-5.78 to -1.22)		-2.40(-4.93 to 0.13)		-2.11 (-4.63 to 0.41)		-2.54 (-4.88 to -0.19)	
Western Pacific	5.21 (-2.80 to 13.22)		3.36 (-4.54 to 11.26)		3.45 (-4.30 to 11.19)		4.67 (-2.96 to 12.30)	
Multi- regional	6.04 (-11.32 to 23.40)		6.92 (-9.80 to 23.64)		7.52 (-9.15 to 24.19)		5.99 (-10.76 to 22.74)	
Recruitment setting		**<0.001**		0.12		0.10		0.26
Community based	Reference		Reference		Reference		Reference	
Hospitals or clinics	**5.73 (3.50 to 7.95)**		2.37 (-1.18 to 5.92)		2.56 (-0.98 to 6.10)		2.09 (-1.44 to 5.62)	
Prisons or residential facility	0.18 (-3.57 to 3.93)		0.31 (-5.56 to 6.18)		0.25 (-5.60 to 6.10)		1.24 (-4.74 to 7.22)	
Workplaces	-0.23 (-4.23 to 3.78)		-2.32 (-8.30 to 3.67)		-2.25(-8.18 to 3.69)		-0.78 (-6.59 to 5.02)	
Internally-displaced persons camps	11.65 (-0.63 to 23.92)		12.97 (-1.03 to 26.96)		13.04(-0.94 to 27.03)		11.96 (-2.05 to 25.97)	
Type of population screened		**<0.001**		0.34		0.38		0.14
General population	Reference		Reference		Reference		Reference	
Contacts	1.68 (-0.92 to 4.29)		-1.15 (-4.42 to 2.13)		-0.96 (-4.23 to 2.31)		0.16 (-3.31 to 3.00)	
PLWH	**7.52 (4.8 to 10.23)**		3.60 (-0.75 to 7.95)		3.34 (-0.97 to 7.64)		**4.85 (0.74 to 8.97)**	
High risk for TB exposure^a^	-0.39 (-3.66 to 2.87)		0.74 (-4.55 to 6.03)		1.02 (-4.26 to 6.31)		-0.62 (-5.92 to 4.67)	
High risk for active TB^b^	2.09 (-1.33 to 5.51)		-0.17 (-4.82 to 5.16)		-0.05 (-5.03 to 4.94)		0.68 (-4.25 to 5.61)	
Population age group		**0.02**		**0.005**		**0.005**		**0.02**
Adults	Reference		Reference		Reference		Reference	
Adults and children	-0.61 (-3.19 to 1.98)		2.33 (-0.78 to 5.45)		2.22 (-0.90 to 5.35)		1.81 (-1.28 to 4.90)	
Children only	**4.13 (0.99 to 7.28)**		**5.71 (2.28 to 9.15)**		**5.61 (2.22 to 8.99)**		**4.76 (1.45 to 8.07)**	
Year of publication		0.58						
2011 to 2016	Reference							
2000 to 2010	1.06 (-1.43 to 3.55)							
1980 to 1999	-0.88 (-4.66 to 2.90)							
Study quality rating		0.97						
High quality	Reference							
Moderate quality	-1.51 (16.82 to 13.79)							
Low quality	0.63 (-7.56 to 8.82)							
Screening modality		**0.01**		0.08				
Symptom screening	Reference		Reference					
CXR	0.14 (-2.97 to 3.24)		1.95 (-1.61 to 5.52)					
Lab Screening^c^	**4.16 (1.60 to 6.71)**		**3.66 (0.68 to 6.64)**					
TST	3.27 (-9.35 to 15.90)		-3.97 (-16.18 to 8.25)					
Diagnostic modality		**0.27**		0.90				
Microscopy	Reference							
Culture &/or GeneXpert MTB/RI	1.68 (-0.87 to 4.22)		-0.40 (-3.02 to 2.22)					
CXR	2.52 (-0.75 to 5.79)		0.21 (-3.00 to 3.42)					
Combined screening and diagnostic algorithm		**0.02**				0.14		
Symptom – Microscopy	Reference				Reference			
Symptom – CXR	2.32 (-1.10 to 5.75)				0.03 (-3.37 to 3.43)			
Symptom – Culture/Xpert	-0.03 (-2.96 to 2.90)				-1.05 (-3.92 to 1.82)			
CXR – Culture/Xpert	0.62 (-3.25 to 4.49)				1.29 (-2.91 to 5.49)			
Lab^c^ – Culture/Xpert	**5.02 (1.74 to 8.30)**				**3.60 (0.08 to 7.28)**			
Other^d^	1.97 (-2.84 to 6.79)				0.17 (-5.04 to 4.70)			
Diagnostic test using GeneXpert MTB/RIF		0.19						0.25
GeneXpert MTB/RIF not used	Reference						Reference	
GeneXpert MTB/RIF used	1.99 (-0.98 to 4.97)						-1.67 (-4.53 to 1.20)	

^a^ High risk for TB exposure: health care worker, prisoner, refugee. ^b^ High risk for active TB: diabetes mellitus, pregnancy, miners. ^c^ Includes screening using AFB smear (94%), culture (71%), or GeneXpert MTB/RIF (8%) ^d^ Other = microscopy for screening and diagnosis (5), CXR for screening and diagnosis (5), CXR for screening then microscopy for diagnosis (2), TST for screening then culture for diagnosis (1), and microscopy for screening then CXR for diagnosis (2).

## Data Availability

Data available within the article or its supplementary materials. The authors confirm that the data supporting the findings of this study are available within the article [and/or] its supplementary materials. All, Share upon request. Data available on request from the authors. The data that support the findings of this study are available from the corresponding author, [RD], upon reasonable request. Basic, Share upon Request.

## Supplementary Materials

Supplementary 1 Supplemental material 1: Systematic Review Search Strategy.

Supplementary 2 Supplemental material 2: List of 224 included studies and raw outcome data.

Supplementary 3 Supplemental Material 3: Table showing Pooled Estimates of Screening Coverage; Based on 256 populations with Data on Numerator and Denominator.

Supplementary 4 Supplemental Material 4: Table showing Meta-regression of Predictors of Coverage of Active TB Cases following Active Case Finding Activities for 256 Unique Populations.

Supplementary 5 Supplemental Material 5: Table Showing Pooled Estimates of TB Yield, Based on 277 Populations with Data on Numerator and Denominator.
