# Supplementary material for: Yield and Coverage of Active Case Finding Interventions for Tuberculosis Control:A Systematic Review and Meta-analysis
Source: Tuberc Res Treat. 2022 Jun 30;2022:9947068. doi: 10.1155/2022/9947068 (PMC9274229; doi:10.1155/2022/9947068)
Supplement: Supplementary 1 — Supplemental material 1: Systematic Review Search Strategy. [file 9947068.f1.docx]

**Search strategy: Yield and Coverage of Active Case Finding Interventions for Tuberculosis Control: Systematic Review and Meta-analysis**

We searched PubMed, EMBASE, Global Health Database, Cochrane Library Central Register of Controlled Trials, Scopus and the WHO Library, using MeSH terms for PubMed and comparable terms for the other databases. We consulted the WHO International Clinical Trials Registry Platform for ongoing trials.

Search terms included “Tuberculosis" OR "Tuberculosis, Pulmonary" OR “Mycobacterium tuberculosis”) AND (“Mass Screening” OR “Contact Tracing” OR “Disease Notification” OR “Epidemiological Monitoring” OR “Disease Transmission, Infectious” OR “contact tracing” OR “case finding” OR “case detection” OR household contact*^[[1]](#footnote-1)^ OR household member* OR “cluster analysis” OR “cluster analyses” OR “contact screening”).

**PubMed strategy #1 conducted on September 18, 2016**

((("Tuberculosis"[Mesh:NoExp] OR "Tuberculosis, Pulmonary"[Mesh] OR “Mycobacterium tuberculosis”[mesh]) AND (“Mass Screening”[mesh] OR “Contact Tracing”[mesh] OR “Disease Notification”[mesh] OR “Epidemiological Monitoring”[mesh] OR “Disease Transmission, Infectious”[mesh] OR “contact tracing” OR “case finding” OR “case detection” OR household contact* OR household member* OR “cluster analysis” OR “cluster analyses” OR “contact screening”) AND ("Mobile Health Units"[Mesh] OR "Ambulatory Care Facilities"[Mesh] OR "House Calls"[Mesh] OR "Residential Facilities"[Mesh] OR "Prisons"[Mesh] OR "Prisoners"[Mesh] OR "Concentration Camps"[Mesh] OR "Homeless Persons"[Mesh] OR "Poverty Areas"[Mesh] OR "Refugees"[Mesh] OR "Transients and Migrants"[Mesh] OR "Workplace"[Mesh] OR "Miners"[Mesh] OR "Mining"[Mesh] OR "Health Personnel"[Mesh] OR "Diabetes Mellitus"[Mesh] OR "hiv clinic" OR "hiv clinics" OR "aids clinic" OR "aids clinics" OR (hiv[ti] AND clinic*[ti]) OR (aids[ti] AND clinic*[ti]) OR “home visits” OR “door to door” OR “house to house” OR mobile campaign* OR mobile clinic* OR mobile hospital* OR “health van” OR “mobile health unit” OR “field based” OR congregate setting* OR nursing home* OR group home* OR orphan* OR ghetto* OR slum OR slums OR undiagnosed) AND ("1980/01/01"[PDat] : "2016/12/31"[PDat])) NOT "review"[Publication Type]) NOT (“animals”[mesh] NOT “humans”[mesh])

**PubMed strategy #2 to capture recent, unindexed references**

(((tuberculosis[ti] OR mycobacterium[ti] OR mycobacteria[ti] OR TB[ti] OR tuberculin[ti]) AND (screen* OR “contact tracing” OR “disease notification” OR “disease transmission” OR “case finding” OR “case detection” OR household contact* OR household member* OR “cluster analysis” OR “cluster analyses” OR “contact screening” OR disease outbreak* OR contact* OR spread*) AND (patient OR patients OR human OR humans OR clinic OR clinics OR clinical* OR community OR communities OR people OR person OR persons OR outpatient* OR workplace* OR camp OR camps OR facility OR facilities OR institution* OR prison* OR jail OR jails OR home OR house* OR orphan* OR ghetto* OR slum OR slums OR worker* OR personnel OR refugee* OR migrant* OR immigrant* OR emigrant* OR miner OR miners OR diabetic* OR hiv OR family OR families OR women OR men OR child OR children OR adolescen* OR “health care” OR health service* OR undiagnosed) AND ("2015”[PDat] : "2016"[PDat])) NOT medline[sb]) NOT “review”[publication type]

1. The asterisk (*) was used as a “wild card” or truncation search feature which provides the ability to search for variant words or spellings employed to build queries [↑](#footnote-ref-1)
