# Supplementary material for: Yield and Coverage of Active Case Finding Interventions for Tuberculosis Control:A Systematic Review and Meta-analysis
Source: Tuberc Res Treat. 2022 Jun 30;2022:9947068. doi: 10.1155/2022/9947068 (PMC9274229; doi:10.1155/2022/9947068)
Supplement: Supplementary 2 — Supplemental material 2: List of 224 included studies and raw outcome data. [file 9947068.f2.docx]

| Article | Year of Study | Study Type | Country | WHO region | Study Quality |
| --- | --- | --- | --- | --- | --- |
| CDC, Prevention [1] | 2003 | Observational-Cross-sectional | Botswana | Africa | Low |
| TB STUDY GROUP [2] | 2013 | Observational-Prospective | India | South East Asia | Moderate |
| Abebe, D. S. [3] | 2011 | Observational-Cross-sectional | Ethiopia | Africa | High |
| Adane, K. [4] | 2016 | Observational-Cross-sectional | Ethiopia | Africa | Moderate |
| Addis, Z. [5] | 2015 | Observational-Cross-sectional | Ethiopia | Africa | High |
| Adelman, M. W. [6] | 2015 | Observational-Cross-sectional | Ethiopia | Africa | High |
| Adetifa, I. M. [7] | 2016 | Surveys | Gambia | Africa | High |
| Ahmad Khan, F. [8] | 2014 | Observational-Cross-sectional | South Africa | Africa | High |
| Akhtar, S. [9] | 2007 | Observational-Cross-sectional | Pakistan | Eastern Mediterranean | High |
| Alemayehu, M. [10] | 2014 | Observational-Cross-sectional | Ethiopia | Africa | High |
| Ali, N. S. [11] | 2002 | Observational-Cross-sectional | Pakistan | Eastern Mediterranean | High |
| Ali, S. [12] | 2015 | Observational-Cross-sectional | Ethiopia | Africa | High |
| Alonso-Echanove, J. [13] | 2001 | Observational-Cross-sectional | Peru | The Americas | High |
| Aluoch, J. A. [14] | 1982 | Observational-Prospective | Kenya | Africa | Moderate |
| Aluoch, J. A. [15] | 1987 | Observational-Cross-sectional | Kenya | Africa | Moderate |
| Aluoch, J. A. [16] | 1985 | Observational-Cross-sectional | Kenya | Africa | Moderate |
| Amare, Deribew [17] | 2012 | Observational-Cross-sectional | Ethiopia | Africa | High |
| Aneja, K. S. [18] | 1980 | Observational-Cross-sectional | India | South East Asia | Moderate |
| Anigilaje, E. A. [19] | 2016 | Retrospective Chart review | Nigeria | Africa | High |
| Arscott-Mills, T. [20] | 2014 | Observational-Cross-sectional | Botswana | Africa | High |
| Assefa, D. [21] | 2011 | Retrospective Chart review | Ethiopia | Africa | High |
| Asuquo, A. E. [22] | 2015 | Observational-Cross-sectional | Nigeria | Africa | High |
| Auld, A. F. [23] | 2016 | Retrospective Chart review | Cote D'Ivoire | Africa | Moderate |
| Ayles, H [24] | 2013 | Quasi RCT | Zambia\South Africa | Africa | High |
| Bakari, M. [25] | 2008 | Observational-Prospective | Tanzania | Africa | High |
| Balasubramanian, R. [26] | 1995 | Observational-Cross-sectional | India | South East Asia | Low |
| Balcha, T. T. [27] | 2014 | Observational-Prospective | Ethiopia | Africa | High |
| Banda, H. T. [28] | 2009 | Observational-Cross-sectional | Malawi | Africa | High |
| Banu, S. [29] | 2013 | Surveys | Bangladesh | South East Asia | High |
| Banu, S. [30] | 2015 | Observational-Cross-sectional | Bangladesh | South East Asia | High |
| Baskaran, Dhanaraj [31] | 2015 | Surveys | India | South East Asia | High |
| Bassett, I. V. [32] | 2016 | RCT | South Africa | Africa | High |
| Basset I. V. [33] | 2010 | Observational-Prospective | South Africa | Africa | High |
| Becerra, M. C. [34] | 2005 | Observational-Cross-sectional | Peru | The Americas | High |
| Beyene, Moges [35] | 2012 | Observational-Cross-sectional | Ethiopia | Africa | High |
| Beyers, N. [36] | 1997 | Observational-Prospective | South Africa | Africa | Moderate |
| Bhat, P. G. [37] | 2013 | Observational-Cross-sectional | India | South East Asia | High |
| Binepal, G. [38] | 2015 | Observational-Cross-sectional | India | South East Asia | Moderate |
| Bjerregaard-Andersen, M. [39] | 2010 | Observational-Cross-sectional | Guinea Bissau | Africa | High |
| Bjerrum, S. [40] | 2016 | Observational-Prospective | Ghana | Africa | High |
| Bloss, E. [41] | 2012 | Observational-Cross-sectional | Botswana | Africa | Moderate |
| Boon, S. den [42] | 2008 | Surveys | South Africa | Africa | High |
| Burmen, B. [43] | 2016 | Retrospective Chart review | Kenya | Africa | High |
| Cadmus, S. I. [44] | 2010 | Observational-Cross-sectional | Nigeria | Africa | High |
| Cassels, A. [45] | 1982 | Observational-Cross-sectional | Nepal | South East Asia | Low |
| Chamie, G. [46] | 2012 | Observational-Cross-sectional | Uganda | Africa | Low |
| TRC, Chennai India [47] | 2011 | Observational-Prospective | India | South East Asia | High |
| Chheng, P. [48] | 2015 | Observational-Cross-sectional | Uganda | Africa | High |
| Chheng, P. [49] | 2008 | Observational-Cross-sectional | Cambodia | South East Asia | High |
| Chisti, M. J. [50] | 2014 | Observational-Prospective | Bangladesh | South East Asia | High |
| Churchyard, G. J [51] | 2011 | RCT | South Africa | Africa | High |
| Churchyard, G. J [52] | 2014 | RCT | South Africa | Africa | High |
| Churchyard, G. J. [53] | 2010 | Observational-Cross-sectional | South Africa | Africa | High |
| Claassens, M. [54] | 2013 | Surveys | South Africa | Africa | High |
| Clarke, M. [55] | 2003 | Observational-Cross-sectional | South Africa | Africa | High |
| Colvin, C. [56] | 2014 | Observational-Cross-sectional | Tanzania | Africa | High |
| Corbett, E. L. [57] | 2010 | RCT | Zimbambwe | Africa | High |
| Corbett, E. L. [58] | 2009 | Surveys | Zimbambwe | Africa | High |
| Corbett, E. L. [59] | 2004 | Observational-Prospective | Zimbambwe | Africa | High |
| Costenaro, P. [60] | 2016 | Observational-Prospective | Uganda | Africa | Moderate |
| Creswell, J. [61] | 2014 | Observational-Cross-sectional | Pakistan | Eastern Mediterranean | High |
| Crook, Am [62] | 2016 | Observational-Prospective | Uganda\Zimbambwe | Africa | High |
| Datiko, D. G. [63] | 2009 | RCT | Ethiopia | Africa | High |
| Day, J. H. [64] | 2006 | Observational-Cross-sectional | South Africa | Africa | High |
| Deery, C. B. [65] | 2014 | Observational-Prospective | South Africa | Africa | High |
| Den Boon, S. [66] | 2006 | Surveys | South Africa | Africa | High |
| Dereje, Habte [67] | 2016 | Observational-Cross-sectional | Ethiopia | Africa | High |
| Deribew, A. [68] | 2011 | Observational-Cross-sectional | Ethiopia | Africa | High |
| Dimairo, M. [69] | 2010 | Observational-Prospective | Zimbambwe | Africa | High |
| Dorman, S. E. [70] | 2012 | Observational-Cross-sectional | South Africa | Africa | High |
| Drain, P. K. [71] | 2014 | Observational-Prospective | South Africa | Africa | High |
| Drain, P. K. [72] | 2015 | Observational-Prospective | South Africa | Africa | High |
| Eang, M. T. [73] | 2012 | Observational-Cross-sectional | Cambodia | South East Asia | High |
| Elden, S. [74] | 2011 | Observational-Prospective | Swaziland | Africa | High |
| Fairall, L. R. [75] | 2005 | RCT | South Africa | Africa | High |
| Fatima, R. [76] | 2014 | Observational-Cross-sectional | Pakistan | South East Asia | High |
| Fox, G. J. [77] | 2012 | Observational-Prospective | Vietnam | South East Asia | High |
| Fuge, T. G. [78] | 2016 | Observational-Cross-sectional | Ethiopia | Africa | Moderate |
| Garcia-Prats, A. J. [79] | 2014 | Observational-Prospective | South Africa | Africa | High |
| Gashu, Z. [80] | 2016 | Observational-Cross-sectional | Ethiopia | Africa | High |
| Gebretsadik, Berhe [81] | 2013 | Surveys | Ethiopia | Africa | High |
| Getahun, H. [82] | 2000 | Observational-Cross-sectional | Ethiopia | Africa | High |
| Ghiya, R. [83] | 2009 | Observational-Cross-sectional | India | South East Asia | High |
| Giri, P. A. [84] | 2013 | Observational-Cross-sectional | India | South East Asia | High |
| Gounder, C. R. [85] | 2011 | Observational-Cross-sectional | South Africa | Africa | High |
| Gounder, C. R. [86] | 2011 | Observational-Cross-sectional | South Africa | Africa | High |
| Govindasamy, D. [87] | 2013 | Observational-Cross-sectional | South Africa | Africa | High |
| Gupta, A. [88] | 2014 | Observational-Cross-sectional | Tanzania | Africa | High |
| Gupta, A [89] | 2007 | Observational-Prospective | India | South East Asia | High |
| Gyawali, N. [90] | 2012 | Observational-Cross-sectional | Nepal | South East Asia | High |
| Habeenzu, C. [91] | 2007 | Observational-Cross-sectional | Zambia | Africa | High |
| Hamusse, S. D. [92] | 2016 | Observational-Cross-sectional | Ethiopia | Africa | High |
| Hanifa, Y. [93] | 2012 | Observational-Prospective | South Africa | Africa | High |
| Hanifa, Y. [94] | 2016 | Observational-Prospective | South Africa | Africa | High |
| Harper, I. [95] | 1996 | Observational-Cross-sectional | Nepal | South East Asia | High |
| Harris, J. B. [96] | 2014 | Observational-Cross-sectional | Zambia | Africa | High |
| Henostroza, G. [97] | 2016 | Observational-Cross-sectional | Zambia | Africa | High |
| Hermans, S. [98] | 2012 | Observational-Prospective | Uganda | Africa | High |
| Ho, J. [99] | 2016 | Observational-Cross-sectional | Vietnam | South East Asia | High |
| Hoffmann, C. J. [100] | 2013 | Observational-Cross-sectional | South Africa | Africa | High |
| Hoog, A. H. van't [101] | 2011 | Observational-Cross-sectional | Kenya | Africa | High |
| Houlihan, C. F. [102] | 2010 | Observational-Cross-sectional | South Africa | Africa | Moderate |
| Iroezindu, M. O. [103] | 2016 | Observational-Cross-sectional | Nigeria | Africa | High |
| Jackson-Sillah, D. [104] | 2007 | Observational-Cross-sectional | Gambia | Africa | High |
| Jaganath, D. [105] | 2013 | Observational-Prospective | Uganda | Africa | High |
| Javaid, A. [106] | 2016 | Observational-Cross-sectional | Pakistan | Eastern Mediterranean | High |
| Jerene, D. [107] | 2015 | Observational-Cross-sectional | Ethiopia | Africa | High |
| John, S. [108] | 2015 | Observational-Cross-sectional | Nigeria | Africa | Moderate |
| Jones-Lopez, E. C. [109] | 2016 | Observational-Prospective | Uganda | Africa | High |
| Joshi, B. [110] | 2015 | Observational-Cross-sectional | Nepal | South East Asia | Moderate |
| Joshi, Y. P. [111] | 2005 | Observational-Cross-sectional | Nepal | South East Asia | High |
| Kali, P. B. N. [112] | 2006 | Observational-Cross-sectional | South Africa | Africa | High |
| Kassim, S. [113] | 2000 | Observational-Cross-sectional | Cote D'Ivoire | Africa | Moderate |
| Khan, A. J. [114] | 2012 | Quasi RCT | Pakistan | South East Asia | High |
| Khanal, S. [115] | 2016 | Observational-Cross-sectional | Nepal | South East Asia | High |
| Khaparde, K. [116] | 2015 | Observational-Cross-sectional | India | South East Asia | High |
| Kimerling, M. E. [117] | 2002 | Observational-Cross-sectional | Cambodia | Western Pacific | High |
| Kliner, M. [118] | 2013 | Observational-Cross-sectional | Swaziland | Africa | Low |
| Koenig, S. P. [119] | 2015 | Observational-Cross-sectional | Haiti | The Americas | Moderate |
| Kranzer, K. [120] | 2012 | Observational-Cross-sectional | South Africa | Africa | High |
| Kufa, T. [121] | 2016 | Observational-Prospective | South Africa | Africa | High |
| Kufa, T. [122] | 2012 | Observational-Cross-sectional | South Africa | Africa | High |
| Kwabla, Mavis Pearl [123] | 2015 | Observational-Cross-sectional | Ghana | Africa | High |
| LaCourse, S. M. [124] | 2014 | Observational-Cross-sectional | Malawi | Africa | High |
| LaCourse, S. M. [125] | 2016 | Observational-Cross-sectional | Kenya | Africa | High |
| Lala, S. G. [126] | 2015 | Observational-Cross-sectional | South Africa | Africa | High |
| Lawn, Stephen D [127] | 2011 | Observational-Cross-sectional | South Africa | Africa | High |
| Lawn, S. D. [128] | 2010 | Observational-Prospective | South Africa | Africa | High |
| Lebina, L. [129] | 2016 | RCT | South Africa | Africa | Moderate |
| Lewis, J. J. [130] | 2009 | Observational-Cross-sectional | South Africa | Africa | High |
| Liaquat, A. [131] | 2015 | Observational-Cross-sectional | Pakistan | Eastern Mediterranean | High |
| Lorent, N. [132] | 2015 | Observational-Prospective | Cambodia | South East Asia | High |
| Lorent, N. [133] | 2014 | Observational-Prospective | Cambodia | South East Asia | High |
| Mabuto, T. [134] | 2015 | Observational-Cross-sectional | South Africa | Africa | Low |
| Madhavi, Pothukuchi [135] | 2011 | Observational-Cross-sectional | India | South East Asia | Low |
| Majumder, A. [136] | 2016 | Observational-Cross-sectional | South Africa | Africa | High |
| Mallikarjun, V. Jali [137] | 2013 | Observational-Prospective | India | South East Asia | Low |
| Manzoor, Shaheena [138] | 2009 | Observational-Cross-sectional | Pakistan | Eastern Mediterranean | High |
| Maritz, E. R. [139] | 2016 | Observational-Prospective | South Africa | Africa | High |
| Martinez, L. [140] | 2016 | Observational-Prospective | Uganda | Africa | Moderate |
| Matee, M. [141] | 2008 | Observational-Prospective | Tanzania | Africa | High |
| McDowell, M. [142] | 2015 | Observational-Cross-sectional | Bangladesh | South East Asia | High |
| Moyo, S. [143] | 2012 | RCT | South Africa | Africa | High |
| Mridul, Gupta [144] | 2016 | Observational-Prospective | India | South East Asia | High |
| Mtwangambate, G. [145] | 2014 | Observational-Prospective | Tanzania | Africa | Low |
| Mugisha, B. [146] | 2006 | Retrospective Chart review | Uganda | Africa | Low |
| Mulenga, H. [147] | 2015 | RCT | South Africa | Africa | High |
| Mupfumi, L [148] | 2014 | RCT | Zimbambwe | Africa | High |
| Musa, B. M. [149] | 2015 | Retrospective Chart review | Nigeria | Africa | High |
| Mushtaque, A. [150] | 1997 | Observational-Cross-sectional | Bangladesh | South East Asia | Low |
| Nachega, J. [151] | 2003 | Observational-Cross-sectional | South Africa | Africa | Low |
| Nair, A. [152] | 2016 | Observational-Cross-sectional | India | South East Asia | High |
| Nair, D. [153] | 2016 | Retrospective Chart review | India | South East Asia | High |
| Nakanjako, D. [154] | 2010 | Observational-Prospective | Uganda | Africa | High |
| Narang, P. [155] | 1992 | Surveys | India | South East Asia | Low |
| Nduba, V. [156] | 2015 | Observational-Prospective | Kenya | Africa | Low |
| Ndwiga, C. [157] | 2013 | Observational-Prospective | Kenya | Africa | Low |
| Ngadaya, E. S. [158] | 2009 | Observational-Cross-sectional | Tanzania | Africa | Low |
| Nguyen, D. T. [159] | 2016 | Observational-Cross-sectional | Vietnam | South East Asia | Low |
| Nliwasa, M. [160] | 2016 | Observational-Prospective | Malawi | Africa | Low |
| Noeske, J. [161] | 2006 | Surveys | Cameroon | Africa | Low |
| Noeske, J. [162] | 2011 | Surveys | Cameroon | Africa | Low |
| Nsanzumuhire, H. [163] | 1981 | Observational-Prospective | Kenya | Africa | Moderate |
| Ntinginya, E. N. [164] | 2012 | Observational-Cross-sectional | Tanzania | Africa | High |
| Nyangulu, D. S. [165] | 1997 | Surveys | Malawi | Africa | Low |
| Ogbudebe, C. L. [166] | 2015 | Observational-Prospective | Nigeria | Africa | Low |
| Okada, K. [167] | 2012 | Observational-Prospective | Cambodia | South East Asia | High |
| Olofin, I. O. [168] | 2016 | RCT | Tanzania | Africa | Moderate |
| Otero, L. [169] | 2016 | Observational-Prospective | Peru | The Americas | Moderate |
| Page-Shipp, L. [170] | 2014 | Observational-Prospective | South Africa | Africa | Low |
| Pathak, R. R. [171] | 2016 | Observational-Prospective | India | South East Asia | Low |
| Prasad, B. M. [172] | 2016 | Observational-Cross-sectional | India | South East Asia | Low |
| Pronyk, P. M. [173] | 2001 | Surveys | South Africa | Africa | Low |
| Puryear, S. [174] | 2013 | Observational-Cross-sectional | Botswana | Africa | High |
| Rangaka, M. X. [175] | 2012 | Observational-Cross-sectional | South Africa | Africa | Low |
| Ray, D. [176] | 1995 | Observational-Prospective | India | South East Asia | Low |
| Reepalu, A. [177] | 2016 | Observational-Cross-sectional | Ethiopia | Africa | Low |
| Rekha Devi, K. [178] | 2013 | Observational-Cross-sectional | India | South East Asia | High |
| Roy, M. [179] | 2016 | Observational-Prospective | Uganda | Africa | High |
| Salazar-Vergara, R. M. L. [180] | 2003 | Observational-Cross-sectional | Philippines | Western Pacific | Low |
| Santha, T. [181] | 2003 | Surveys | India | South East Asia | Low |
| Schalkwyk, C. van [182] | 2014 | Observational-Prospective | South Africa | Africa | High |
| Seddon, J. A. [183] | 2013 | Observational-Cross-sectional | South Africa | Africa | Low |
| Sekandi, J. N. [184] | 2014 | Observational-Cross-sectional | Uganda | Africa | Low |
| Sekandi, J. N. [185] | 2009 | Surveys | Uganda | Africa | Low |
| Shabbir, Iffat [186] | 2009 | Observational-Cross-sectional | Pakistan | Eastern Mediterranean | Low |
| Shah, S. A. [187] | 2013 | Observational-Cross-sectional | Pakistan | Eastern Mediterranean | Low |
| Shah, S. [188] | 2009 | Observational-Cross-sectional | Ethiopia | Africa | Low |
| Shapiro, A. E. [189] | 2012 | Observational-Cross-sectional | South Africa | Africa | Low |
| Shargie, E. B. [190] | 2006 | RCT | Ethiopia | Africa | Low |
| Sharma, S. K. [191] | 2015 | Observational-Cross-sectional | India | South East Asia | Low |
| Shayo, G. A. [192] | 2014 | Observational-Cross-sectional | Tanzania | Africa | Low |
| Shrivastava, S. R. [193] | 2013 | Surveys | India | South East Asia | Low |
| Sia, I. G. [194] | 2010 | Observational-Cross-sectional | Philippines | Western Pacific | Low |
| Sinfield, R. [195] | 2006 | Observational-Prospective | Malawi | Africa | Low |
| Sretrirutchai, S. [196] | 2002 | Observational-Cross-sectional | Thailand | South East Asia | Low |
| Srichand, Batra [197] | 2012 | Retrospective Chart review | Pakistan | Eastern Mediterranean | High |
| Ssemmondo, E. [198] | 2016 | RCT | Uganda | Africa | Low |
| Suresh, S. [199] | 2016 | Retrospective Chart review | India | South East Asia | Moderate |
| Swindells, S. [200] | 2013 | Observational-Cross-sectional | Multi | Multiple | Low |
| Szkwarko, D. [201] | 2016 | Observational-Cross-sectional | Kenya | Africa | Low |
| Tadesse, T. [202] | 2011 | Observational-Cross-sectional | Ethiopia | Africa | High |
| Tadesse, T. [203] | 2013 | Observational-Prospective | Ethiopia | Africa | High |
| Tamhane, A. [204] | 2009 | Retrospective Chart review | Cambodia | Western Pacific | Low |
| Telisinghe, L. [205] | 2014 | Surveys | South Africa | Africa | Low |
| Thind, D. [206] | 2012 | Observational-Prospective | South Africa | Africa | Low |
| Tiam, A. [207] | 2014 | Observational-Prospective | Lesotho | Africa | Moderate |
| Topley, J. M. [208] | 1996 | Observational-Cross-sectional | Malawi | Africa | Low |
| Triasih, R. [209] | 2015 | Observational-Prospective | Indonesia | South East Asia | Moderate |
| Trinh, T. T. [210] | 2015 | Observational-Cross-sectional | Vietnam | South East Asia | Low |
| Tupasi, T. E. [211] | 2000 | Surveys | Philippines | Western Pacific | Low |
| Vree, M. [212] | 2007 | Surveys | Vietnam | South East Asia | High |
| Waako, J. [213] | 2013 | Observational-Prospective | Uganda | Africa | Low |
| Wares, D. F. [214] | 2000 | Retrospective Chart review | Nepal | Eastern Mediterranean | Low |
| Whalen, C. C. [215] | 2011 | Observational-Prospective | Uganda | Africa | High |
| Woldesemayat, E. M. [216] | 2015 | Observational-Prospective | Ethiopia | Africa | Low |
| Wood, R. [217] | 2007 | Observational-Cross-sectional | South Africa | Africa | Low |
| Wyk, S. S. van [218] | 2012 | Observational-Cross-sectional | South Africa | Africa | High |
| Xavier, T. [219] | 1992 | Observational-Cross-sectional | India | South East Asia | Low |
| Yared, Tadesse [220] | 2016 | Observational-Cross-sectional | Ethiopia | Africa | Low |
| Yassin, M. A. [221] | 2013 | Observational-Cross-sectional | Ethiopia | Africa | Low |
| Yimer, S. [222] | 2009 | Observational-Cross-sectional | Ethiopia | Africa | Low |
| Zachariah, R. [223] | 2003 | Observational-Cross-sectional | Malawi | Africa | Low |
| Zaeh, S. [224] | 2013 | Observational-Prospective | Ethiopia | Africa | High |

**Supplemental raw data of yield and coverage in 277 unique populations**

| **Author** | **Year** | **Number of new TB cases** | **Number targeted** | **Number screened** | **Percent Yield** | **Percent Coverage** | **Quality Rating** |
| --- | --- | --- | --- | --- | --- | --- | --- |
| Centers for Disease, Control Prevention | 2003 | 2 | 288 | 263 | 0.76 | 91.32 | Low quality |
| Centers for Disease, Control Prevention | 2003 | 19 | 1,173 | 1,027 | 1.85 | 87.55 | Low quality |
| TB STUDY GROUP | 2013 | 18 | 31,146 | 13,961 | 0.13 | 44.82 | Moderate quality |
| Abebe, D. S. | 2011 | 33 | 2,300 | 2,300 | 1.43 | 100.00 | High quality |
| Adane, K. | 2016 | 844 | 9,299 | 9,299 | 9.08 | 100.00 | Moderate quality |
| Addis, Z. | 2015 | 33 | 1,624 | 1,624 | 2.03 | 100.00 | High quality |
| Adelman, M. W. | 2015 | 13 | 828 | 828 | 1.57 | 100.00 | High quality |
| Adetifa, I. M. | 2016 | 73 | 43,100 | 43,100 | 0.17 | 100.00 | High quality |
| Ahmad Khan, F. | 2014 | 65 | 825 | 737 | 8.82 | 89.33 | High quality |
| Akhtar, S. | 2007 | 18 | 6,083 | 5,479 | 0.33 | 90.07 | High quality |
| Alemayehu, M. | 2014 | 15 | 250 | 250 | 6.00 | 100.00 | High quality |
| Ali, N. S. | 2002 | 1 | 207 | 207 | 0.48 | 100.00 | High quality |
| Ali, S. | 2015 | 20 | 15,495 | 15,495 | 0.13 | 100.00 | High quality |
| Alonso-Echanove, J. | 2001 | 36 | 2,300 | 1,600 | 2.25 | 69.57 | High quality |
| Aluoch, J. A. | 1982 | 26 | 19,605 | 980 | 2.65 | 5.00 | Moderate quality |
| Aluoch, J. A. | 1987 | 8 | 342 | 261 | 3.07 | 76.32 | Moderate quality |
| Aluoch, J. A. | 1985 | 109 | 118,847 | 87,845 | 0.12 | 73.91 | Moderate quality |
| Amare, Deribew | 2012 | 17 | 30,040 | 27,597 | 0.06 | 91.87 | High quality |
| Aneja, K. S. | 1980 | 38 | 29,260 | 29,260 | 0.13 | 100.00 | Moderate quality |
| Aneja, K. S. | 1980 | 12 | 29,833 | 29,833 | 0.04 | 100.00 | Moderate quality |
| Aneja, K. S. | 1980 | 24 | 33,377 | 33,377 | 0.07 | 100.00 | Moderate quality |
| Anigilaje, E. A. | 2016 | 113 | 408 | 368 | 30.71 | 90.20 | High quality |
| Arscott-Mills, T. | 2014 | 6 | 176 | 118 | 5.08 | 67.05 | High quality |
| Assefa, D. | 2011 | 34 | 300 | 300 | 11.33 | 100.00 | High quality |
| Asuquo, A. E. | 2015 | 374 | 9,251 | 9,251 | 4.04 | 100.00 | High quality |
| Auld, A. F. | 2016 | 111 | 3,609 | 1,263 | 8.79 | 35.00 | Moderate quality |
| Ayles, H | 2013 | 214 | 30,315 | 16,253 | 1.32 | 53.61 | High quality |
| Ayles, H | 2013 | 276 | 32,598 | 17,170 | 1.61 | 52.67 | High quality |
| Ayles, H | 2013 | 175 | 29,341 | 14,204 | 1.23 | 48.41 | High quality |
| Ayles, H | 2013 | 229 | 31,536 | 16,836 | 1.36 | 53.39 | High quality |
| Bakari, M. | 2008 | 136 | 1,176 | 1,176 | 11.56 | 100.00 | High quality |
| Balasubramanian, R. | 1995 | 12 | 5,755 | 5,755 | 0.21 | 100.00 | Low quality |
| Balcha, T. T. | 2014 | 145 | 873 | 812 | 17.86 | 93.01 | High quality |
| Banda, H. T. | 2009 | 52 | 7,661 | 7,661 | 0.68 | 100.00 | High quality |
| Banu, S. | 2013 | 1 | 5,151 | 5,146 | 0.02 | 99.90 | High quality |
| Banu, S. | 2013 | 25 | - | 9,877 | 0.25 | - | High quality |
| Banu, S. | 2015 | 422 | 18,218 | 18,218 | 2.32 | 100.00 | High quality |
| Banu, S. | 2015 | 44 | 42,367 | 42,367 | 0.10 | 100.00 | High quality |
| Baskaran, Dhanaraj | 2015 | 170 | 59,957 | 55,617 | 0.31 | 92.76 | High quality |
| Bassett, I. V. | 2016 | 523 | 1,899 | 1,685 | 31.04 | 88.73 | High quality |
| Bassett, I. V. | 2010 | 158 | 1,036 | 825 | 19.15 | 79.63 | High quality |
| Becerra, M. C. | 2005 | 3 | 2,253 | 2,253 | 0.13 | 100.00 | High quality |
| Becerra, M. C. | 2005 | 8 | 1,094 | 1,094 | 0.73 | 100.00 | High quality |
| Beyene, Moges | 2012 | 26 | 1,754 | 1,754 | 1.48 | 100.00 | High quality |
| Beyers, N. | 1997 | 52 | 171 | 155 | 33.55 | 90.64 | Moderate quality |
| Beyers, N. | 1997 | 33 | 493 | 154 | 21.43 | 31.24 | Moderate quality |
| Bhat, P. G. | 2013 | 19 | 1,927 | 1,173 | 1.62 | 60.87 | High quality |
| Binepal, G. | 2015 | 58 | 8,346 | 8,346 | 0.69 | 100.00 | Moderate quality |
| Bjerregaard-Andersen, M. | 2010 | 2 | 3,714 | 2,989 | 0.07 | 80.48 | High quality |
| Bjerregaard-Andersen, M. | 2010 | 0 | 718 | 571 | 0.00 | 79.53 | High quality |
| Bjerrum, S. | 2016 | 60 | 571 | 498 | 12.05 | 87.22 | High quality |
| Bloss, E. | 2012 | 19 | 11,799 | 11,799 | 0.16 | 100.00 | Moderate quality |
| Boon, S. den | 2008 | 27 | 3,971 | 3,483 | 0.78 | 87.71 | High quality |
| Burmen, B. | 2016 | 26 | 1,020 | 995 | 2.61 | 97.55 | High quality |
| Cadmus, S. I. | 2010 | 10 | 101 | 78 | 12.82 | 77.23 | High quality |
| Cassels, A. | 1982 | 111 | 67,068 | 67,068 | 0.17 | 100.00 | Low quality |
| Chamie, G. | 2012 | 0 | 3,150 | 2,323 | 0.00 | 73.75 | Low quality |
| Chamie, G. | 2012 | 0 | 3,150 | 2,020 | 0.00 | 64.13 | Low quality |
| Chennai, | 2011 | 3,777 | 246,845 | 246,845 | 1.53 | 100.00 | High quality |
| Chennai, | 2011 | 22 | 779 | 779 | 2.82 | 100.00 | High quality |
| Chennai, | 2011 | 143 | 5,562 | 5,562 | 2.57 | 100.00 | High quality |
| Chheng, P. | 2015 | 13 | 141 | 141 | 9.22 | 100.00 | High quality |
| Chheng, P. | 2015 | 5 | 54 | 54 | 9.26 | 100.00 | High quality |
| Chheng, P. | 2008 | 29 | 583 | 496 | 5.85 | 85.08 | High quality |
| Chisti, M. J. | 2014 | 27 | 405 | 396 | 6.82 | 97.78 | High quality |
| Churchyard, Gj | 2011 | 670 | 11,317 | 10,997 | 6.09 | 97.17 | High quality |
| Churchyard, Gj | 2011 | 632 | 11,317 | 11,015 | 5.74 | 97.33 | High quality |
| Churchyard, Gj | 2014 | 887 | 40,981 | 31,980 | 2.77 | 78.04 | High quality |
| Churchyard, Gj | 2014 | 119 | 5,809 | 5,557 | 2.14 | 95.66 | High quality |
| Churchyard, Gj | 2014 | 166 | 7,225 | 7,049 | 2.35 | 97.56 | High quality |
| Churchyard, Gj | 2014 | 856 | 37,763 | 31,194 | 2.74 | 82.60 | High quality |
| Churchyard, G. J. | 2010 | 360 | 23,299 | 23,286 | 1.55 | 99.94 | High quality |
| Claassens, M. | 2013 | 146 | 6,296 | 6,262 | 2.33 | 99.46 | High quality |
| Clarke, M. | 2003 | 6 | 422 | 356 | 1.69 | 84.36 | High quality |
| Colvin, C. | 2014 | 104 | 434 | 419 | 24.82 | 96.54 | High quality |
| Colvin, C. | 2014 | 17 | 178 | 178 | 9.55 | 100.00 | High quality |
| Corbett, El | 2010 | 255 | 5,466 | 5,466 | 4.67 | 100.00 | High quality |
| Corbett, El | 2010 | 137 | 4,721 | 4,711 | 2.91 | 99.79 | High quality |
| Corbett, E. L. | 2009 | 91 | 12,426 | 10,092 | 0.90 | 81.22 | High quality |
| Corbett, E. L. | 2004 | 64 | 1,687 | 1,687 | 3.79 | 100.00 | High quality |
| Corbett, E. L. | 2004 | 45 | 1,773 | 1,734 | 2.60 | 97.80 | High quality |
| Costenaro, P. | 2016 | 36 | 899 | 529 | 6.81 | 58.84 | Moderate quality |
| Creswell, J. | 2014 | 1,010 | 541,336 | 529,447 | 0.19 | 97.80 | High quality |
| Crook, Am | 2016 | 69 | 969 | 969 | 7.12 | 100.00 | High quality |
| Datiko, D. G. | 2009 | 230 | 178,138 | 178,138 | 0.13 | 100.00 | High quality |
| Day, J. H. | 2006 | 44 | 1,310 | 1,085 | 4.06 | 82.82 | High quality |
| Deery, C. B. | 2014 | 2 | 267 | 267 | 0.75 | 100.00 | High quality |
| Den Boon, S. | 2006 | 29 | 3,483 | 2,608 | 1.11 | 74.88 | High quality |
| Dereje, Habte | 2016 | 14 | 393 | 353 | 3.97 | 89.82 | High quality |
| Deribew, A. | 2011 | 233 | 545 | 506 | 46.05 | 92.84 | High quality |
| Dimairo, M. | 2010 | 218 | 1,234 | 1,195 | 18.24 | 96.84 | High quality |
| Dorman, S. E. | 2012 | 187 | 7,346 | 7,331 | 2.55 | 99.80 | High quality |
| Drain, P. K. | 2014 | 60 | 411 | 342 | 17.54 | 83.21 | High quality |
| Drain, P. K. | 2015 | 54 | 351 | 320 | 16.88 | 91.17 | High quality |
| Eang, M. T. | 2012 | 405 | 33,631 | 33,631 | 1.20 | 100.00 | High quality |
| Elden, S. | 2011 | 18 | 1,129 | 1,129 | 1.59 | 100.00 | High quality |
| Elden, S. | 2011 | 10 | 338 | 338 | 2.96 | 100.00 | High quality |
| Fairall, L. R. | 2005 | 57 | 1,000 | 892 | 6.39 | 89.20 | High quality |
| Fairall, L. R. | 2005 | 34 | 999 | 890 | 3.82 | 89.09 | High quality |
| Fatima, R. | 2014 | 3,117 | 165,280 | 165,280 | 1.89 | 100.00 | High quality |
| Fox, G. J. | 2012 | 5 | 657 | 545 | 0.92 | 82.95 | High quality |
| Fuge, T. G. | 2016 | 3 | 859 | 859 | 0.35 | 100.00 | Moderate quality |
| Garcia-Prats, A. J. | 2014 | - | 38 | 32 | 0.00 | 84.21 | High quality |
| Gashu, Z. | 2016 | 2,091 | 272,515 | 272,441 | 0.77 | 99.97 | High quality |
| Gebretsadik, Berhe | 2013 | 16 | 12,175 | 12,175 | 0.13 | 100.00 | High quality |
| Getahun, H. | 2000 | 117 | - | 181 | 64.64 | - | High quality |
| Ghiya, R. | 2009 | 211 | 500 | 500 | 42.20 | 100.00 | High quality |
| Giri, P. A. | 2013 | 172 | 1,012 | 1,012 | 17.00 | 100.00 | High quality |
| Gounder, C. R. | 2011 | 30 | 443 | 422 | 7.11 | 95.26 | High quality |
| Gounder, C. R. | 2011 | 15 | 8,862 | 8,862 | 0.17 | 100.00 | High quality |
| Govindasamy, D. | 2013 | 14 | - | 9,806 | 0.14 | - | High quality |
| Gupta, A. | 2014 | 6 | 222 | 150 | 4.00 | 67.57 | High quality |
| Gupta, A | 2007 | 24 | 715 | 715 | 3.36 | 100.00 | High quality |
| Gyawali, N. | 2012 | 13 | 802 | 802 | 1.62 | 100.00 | High quality |
| Habeenzu, C. | 2007 | 245 | 6,118 | 1,080 | 22.69 | 17.65 | High quality |
| Hamusse, S. D. | 2016 | 106 | 33,073 | 33,073 | 0.32 | 100.00 | High quality |
| Hanifa, Y. | 2012 | 114 | 361 | 361 | 31.58 | 100.00 | High quality |
| Hanifa, Y. | 2016 | 56 | 586 | 424 | 13.21 | 72.35 | High quality |
| Harper, I. | 1996 | 71 | 4,009 | 4,009 | 1.77 | 100.00 | High quality |
| Harris, J. B. | 2014 | 62 | 1,801 | 1,801 | 3.44 | 100.00 | High quality |
| Henostroza, G. | 2016 | 72 | 502 | 399 | 18.05 | 79.48 | High quality |
| Hermans, S. | 2012 | 180 | 10,525 | 10,525 | 1.71 | 100.00 | High quality |
| Ho, J. | 2016 | 169 | 51,136 | 43,435 | 0.39 | 84.94 | High quality |
| Hoffmann, C. J. | 2013 | 35 | 1,515 | 1,451 | 2.41 | 95.78 | High quality |
| Hoog, A. H. van't | 2011 | 123 | 20,710 | 20,566 | 0.60 | 99.30 | High quality |
| Houlihan, C. F. | 2010 | 81 | 801 | 583 | 13.89 | 72.78 | Moderate quality |
| Iroezindu, M. O. | 2016 | 26 | 354 | 339 | 7.67 | 95.76 | High quality |
| Jackson-Sillah, D. | 2007 | 33 | 2,381 | 2,381 | 1.39 | 100.00 | High quality |
| Jaganath, D. | 2013 | 79 | 766 | 761 | 10.38 | 99.35 | High quality |
| Javaid, A. | 2016 | 51 | 610 | 610 | 8.36 | 100.00 | High quality |
| Jerene, D. | 2015 | 389 | 16,512 | 15,527 | 2.51 | 94.03 | High quality |
| John, S. | 2015 | 1,310 | 96,376 | 96,376 | 1.36 | 100.00 | Moderate quality |
| Jones-Lopez, E. C. | 2016 | 8 | 460 | 369 | 2.17 | 80.22 | High quality |
| Joshi, B. | 2015 | 51 | - | 2,107 | 2.42 | - | Moderate quality |
| Joshi, B. | 2015 | 106 | - | 11,421 | 0.93 | - | Moderate quality |
| Joshi, B. | 2015 | - | - | 700 | 0.00 | - | Moderate quality |
| Joshi, B. | 2015 | - | - | 811 | 0.00 | - | Moderate quality |
| Joshi, B. | 2015 | 3 | - | 602 | 0.50 | - | Moderate quality |
| Joshi, Y. P. | 2005 | 10 | 125 | 125 | 8.00 | 100.00 | High quality |
| Kali, P. B. N. | 2006 | 8 | 549 | 370 | 2.16 | 67.40 | High quality |
| Kassim, S. | 2000 | 5 | 643 | 512 | 0.98 | 79.63 | Moderate quality |
| Khan, A. J. | 2012 | 273 | - | 81,700 | 0.33 | - | High quality |
| Khan, A. J. | 2012 | 603 | - | 388,196 | 0.16 | - | High quality |
| Khanal, S. | 2016 | 523 | - | 103,027 | 0.51 | - | High quality |
| Khanal, S. | 2016 | 21 | - | 5,490 | 0.38 | - | High quality |
| Khanal, S. | 2016 | 24 | - | 7,424 | 0.32 | - | High quality |
| Khanal, S. | 2016 | 36 | - | 12,023 | 0.30 | - | High quality |
| Khanal, S. | 2016 | 1 | - | 1,019 | 0.10 | - | High quality |
| Khanal, S. | 2016 | 130 | - | 2,149 | 6.05 | - | High quality |
| Khanal, S. | 2016 | 503 | - | 14,547 | 3.46 | - | High quality |
| Khaparde, K. | 2015 | 17 | 1,556 | 1,490 | 1.14 | 95.76 | High quality |
| Kimerling, M. E. | 2002 | 40 | 787 | 441 | 9.07 | 56.04 | High quality |
| Kliner, M. | 2013 | 2 | 111 | 26 | 7.69 | 23.42 | Low quality |
| Kliner, M. | 2013 | 4 | 658 | 157 | 2.55 | 23.86 | Low quality |
| Koenig, S. P. | 2015 | 233 | - | 1,420 | 16.41 | - | Moderate quality |
| Koenig, S. P. | 2015 | 34 | - | 282 | 12.06 | - | Moderate quality |
| Kranzer, K. | 2012 | 56 | 1,385 | 1,130 | 4.96 | 81.59 | High quality |
| Kufa, T. | 2016 | 15 | 839 | 634 | 2.37 | 75.57 | High quality |
| Kufa, T. | 2012 | 50 | 443 | 422 | 11.85 | 95.26 | High quality |
| Kwabla, Mavis Pearl | 2015 | 1 | 431 | 389 | 0.26 | 90.26 | High quality |
| LaCourse, S. M. | 2014 | 219 | 300 | 300 | 73.00 | 100.00 | High quality |
| LaCourse, S. M. | 2016 | 7 | 388 | 288 | 2.43 | 74.23 | High quality |
| Lala, S. G. | 2015 | 43 | 1,341 | 1,285 | 3.35 | 95.82 | High quality |
| Lala, S. G. | 2015 | 23 | 576 | 541 | 4.25 | 93.92 | High quality |
| Lawn, Stephen D | 2011 | 81 | 515 | 468 | 17.31 | 90.87 | High quality |
| Lawn, S. D. | 2010 | 87 | 241 | 241 | 36.10 | 100.00 | High quality |
| Lebina, L. | 2016 | 23 | - | 863 | 2.67 | - | Moderate quality |
| Lebina, L. | 2016 | 8 | - | 515 | 1.55 | - | Moderate quality |
| Lewis, J. J. | 2009 | 51 | 2,240 | 1,978 | 2.58 | 88.30 | High quality |
| Liaquat, A. | 2015 | 3 | 80 | 80 | 3.75 | 100.00 | High quality |
| Lorent, N. | 2015 | 278 | 4,616 | 4,606 | 6.04 | 99.78 | High quality |
| Lorent, N. | 2014 | 783 | 346,000 | 315,874 | 0.25 | 91.29 | High quality |
| Mabuto, T. | 2015 | 10 | 1,600 | 686 | 1.46 | 42.88 | Low quality |
| Madhavi, Pothukuchi | 2011 | - | 172 | 116 | 0.00 | 67.44 | Low quality |
| Majumder, A. | 2016 | - | 672 | 672 | 0.00 | 100.00 | High quality |
| Mallikarjun, V. Jali | 2013 | 2 | 4,118 | 2,072 | 0.10 | 50.32 | Low quality |
| Manzoor, Shaheena | 2009 | - | 261 | 261 | 0.00 | 100.00 | High quality |
| Maritz, E. R. | 2016 | 128 | 1,351 | 1,351 | 9.47 | 100.00 | High quality |
| Martinez, L. | 2016 | 16 | 915 | 915 | 1.75 | 100.00 | Moderate quality |
| Martinez, L. | 2016 | 18 | 1,018 | 1,018 | 1.77 | 100.00 | Moderate quality |
| Matee, M. | 2008 | 103 | 2,216 | 2,216 | 4.65 | 100.00 | High quality |
| McDowell, M. | 2015 | 1,587 | 32,587 | 24,779 | 6.40 | 76.04 | High quality |
| Moyo, S. | 2012 | 178 | 2,392 | 1,977 | 9.00 | 82.65 | High quality |
| Moyo, S. | 2012 | 72 | 2,394 | 1,952 | 3.69 | 81.54 | High quality |
| Mridul, Gupta | 2016 | 6 | 521 | 521 | 1.15 | 100.00 | High quality |
| Mtwangambate, G. | 2014 | 7 | 700 | 693 | 1.01 | 99.00 | Low quality |
| Mugisha, B. | 2006 | 52 | 243 | 243 | 21.40 | 100.00 | Low quality |
| Mugisha, B. | 2006 | 293 | 7,453 | 6,305 | 4.65 | 84.60 | Low quality |
| Mulenga, H. | 2015 | 169 | 1,017 | 1,017 | 16.62 | 100.00 | High quality |
| Mupfumi, L | 2014 | 21 | 210 | 172 | 12.21 | 81.90 | High quality |
| Mupfumi, L | 2014 | 17 | 214 | 182 | 9.34 | 85.05 | High quality |
| Musa, B. M. | 2015 | 47 | 345 | 345 | 13.62 | 100.00 | High quality |
| Mushtaque, A. | 1997 | 13 | 8,720 | 8,720 | 0.15 | 100.00 | Low quality |
| Mushtaque, A. | 1997 | 5 | 7,146 | 7,146 | 0.07 | 100.00 | Low quality |
| Mushtaque, A. | 1997 | 6 | 8,639 | 8,639 | 0.07 | 100.00 | Low quality |
| Nachega, J. | 2003 | 13 | 438 | 318 | 4.09 | 72.60 | Low quality |
| Nair, A. | 2016 | 16 | 152 | 151 | 10.60 | 99.34 | High quality |
| Nair, D. | 2016 | 29 | 643 | 544 | 5.33 | 84.60 | High quality |
| Nakanjako, D. | 2010 | 749 | 10,924 | 10,767 | 6.96 | 98.56 | High quality |
| Narang, P. | 1992 | 1,252 | 825,606 | 773,493 | 0.16 | 93.69 | Low quality |
| Nduba, V. | 2015 | 34 | 5,541 | 5,004 | 0.68 | 90.31 | Low quality |
| Ndwiga, C. | 2013 | 15 | 13,332 | 12,932 | 0.12 | 97.00 | Low quality |
| Ngadaya, E. S. | 2009 | 271 | 65,530 | 65,530 | 0.41 | 100.00 | Low quality |
| Nguyen, D. T. | 2016 | 28 | 639 | 397 | 7.05 | 62.13 | Low quality |
| Nliwasa, M. | 2016 | 56 | 773 | 773 | 7.24 | 100.00 | Low quality |
| noeske, J. | 2006 | 60 | 2,830 | 2,474 | 2.43 | 87.42 | Low quality |
| noeske, J. | 2011 | 40 | 3,779 | 3,219 | 1.24 | 85.18 | Low quality |
| Nsanzumuhire, H. | 1981 | 16 | 24,499 | 1,293 | 1.24 | 5.28 | Moderate quality |
| Ntinginya, E. N. | 2012 | 5 | 219 | 219 | 2.28 | 100.00 | High quality |
| Nyangulu, D. S. | 1997 | 33 | 1,315 | 914 | 3.61 | 69.51 | Low quality |
| Ogbudebe, C. L. | 2015 | 1,079 | 16,743 | 16,743 | 6.44 | 100.00 | Low quality |
| Okada, K. | 2012 | 100 | 1,753 | 1,423 | 7.03 | 81.18 | High quality |
| Okada, K. | 2012 | 271 | 22,160 | 22,160 | 1.22 | 100.00 | High quality |
| Olofin, I. O. | 2016 | 183 | 2,387 | 2,358 | 7.76 | 98.79 | Moderate quality |
| Otero, L. | 2016 | 205 | 5,496 | 5,466 | 3.75 | 99.45 | Moderate quality |
| Page-Shipp, L. | 2014 | 12 | 15,000 | 1,661 | 0.72 | 11.07 | Low quality |
| Pathak, R. R. | 2016 | 39 | 440 | 365 | 10.68 | 82.95 | Low quality |
| Prasad, B. M. | 2016 | 14,447 | 19,946,114 | 19,946,114 | 0.07 | 100.00 | Low quality |
| Pronyk, P. M. | 2001 | 6 | 38,251 | 38,127 | 0.02 | 99.68 | Low quality |
| Puryear, S. | 2013 | 12 | 548 | 548 | 2.19 | 100.00 | High quality |
| Rangaka, M. X. | 2012 | 126 | 2,136 | 2,090 | 6.03 | 97.85 | Low quality |
| Ray, D. | 1995 | 22 | 23,000 | 19,129 | 0.12 | 83.17 | Low quality |
| Ray, D. | 1995 | 21 | 23,000 | 19,570 | 0.11 | 85.09 | Low quality |
| Reepalu, A. | 2016 | 158 | 886 | 812 | 19.46 | 91.65 | Low quality |
| Rekha Devi, K. | 2013 | 417 | 2,961 | 2,961 | 14.08 | 100.00 | High quality |
| Rekha Devi, K. | 2013 | 110 | 1,410 | 1,410 | 7.80 | 100.00 | High quality |
| Roy, M. | 2016 | 12 | 2,613 | 2,439 | 0.49 | 93.34 | High quality |
| Salazar-Vergara, R. M. L. | 2003 | 5 | 166 | 153 | 3.27 | 92.17 | Low quality |
| Santha, T. | 2003 | 216 | 36,075 | 32,663 | 0.66 | 90.54 | Low quality |
| Schalkwyk, C. van | 2014 | 26 | 2,377 | 2,089 | 1.24 | 87.88 | High quality |
| Seddon, J. A. | 2013 | 15 | 281 | 228 | 6.58 | 81.14 | Low quality |
| Sekandi, J. N. | 2014 | 39 | 7,391 | 5,102 | 0.76 | 69.03 | Low quality |
| Sekandi, J. N. | 2009 | 33 | 1,000 | 930 | 3.55 | 93.00 | Low quality |
| Shabbir, Iffat | 2009 | 12 | 4,580 | 4,580 | 0.26 | 100.00 | Low quality |
| Shah, S. A. | 2013 | 490 | 19,191 | 19,191 | 2.55 | 100.00 | Low quality |
| Shah, S. | 2009 | 32 | 498 | 438 | 7.31 | 87.95 | Low quality |
| Shapiro, A. E. | 2012 | 4 | 983 | 785 | 0.51 | 79.86 | Low quality |
| Shapiro, A. E. | 2012 | 169 | 2,843 | 2,166 | 7.80 | 76.19 | Low quality |
| Shargie, E. B. | 2006 | 159 | 127,607 | 127,607 | 0.12 | 100.00 | Low quality |
| Sharma, S. K. | 2015 | 100 | 105,202 | 98,599 | 0.10 | 93.72 | Low quality |
| Shayo, G. A. | 2014 | 14 | 474 | 373 | 3.75 | 78.69 | Low quality |
| Shrivastava, S. R. | 2013 | 33 | 529,452 | 529,452 | 0.01 | 100.00 | Low quality |
| Sia, I. G. | 2010 | 113 | 1,086 | 897 | 12.60 | 82.60 | Low quality |
| Sinfield, R. | 2006 | 44 | 285 | 195 | 22.56 | 68.42 | Low quality |
| Sretrirutchai, S. | 2002 | 27 | 4,751 | 4,751 | 0.57 | 100.00 | Low quality |
| Srichand, Batra | 2012 | 121 | 6,613 | 6,613 | 1.83 | 100.00 | High quality |
| Ssemmondo, E. | 2016 | 9 | 36,785 | 27,214 | 0.03 | 73.98 | Low quality |
| Suresh, S. | 2016 | 17 | 11,617 | 11,617 | 0.15 | 100.00 | Moderate quality |
| Swindells, S. | 2013 | 85 | 816 | 707 | 12.02 | 86.64 | Low quality |
| Szkwarko, D. | 2016 | 1 | 1,000 | 116 | 0.86 | 11.60 | Low quality |
| Tadesse, T. | 2011 | 41 | 23,590 | 23,590 | 0.17 | 100.00 | High quality |
| Tadesse, T. | 2013 | 74 | 23,794 | 23,794 | 0.31 | 100.00 | High quality |
| Tamhane, A. | 2009 | 244 | 1,261 | 881 | 27.70 | 69.87 | Low quality |
| Telisinghe, L. | 2014 | 73 | 1,046 | 981 | 7.44 | 93.79 | Low quality |
| Thind, D. | 2012 | 93 | 3,033 | 3,033 | 3.07 | 100.00 | Low quality |
| Thind, D. | 2012 | 50 | 411 | 361 | 13.85 | 87.83 | Low quality |
| Tiam, A. | 2014 | 2 | 160 | 158 | 1.27 | 98.75 | Moderate quality |
| Tiam, A. | 2014 | 1 | 640 | 640 | 0.16 | 100.00 | Moderate quality |
| Topley, J. M. | 1996 | 180 | 282 | 282 | 63.83 | 100.00 | Low quality |
| Triasih, R. | 2015 | 25 | 269 | 269 | 9.29 | 100.00 | Moderate quality |
| Trinh, T. T. | 2015 | 17 | 1,281 | 850 | 2.00 | 66.35 | Low quality |
| Tupasi, T. E. | 2000 | 174 | 14,656 | 13,001 | 1.34 | 88.71 | Low quality |
| Vree, M. | 2007 | 16 | 68,946 | 68,946 | 0.02 | 100.00 | High quality |
| Waako, J. | 2013 | 23 | 8,000 | 5,000 | 0.46 | 62.50 | Low quality |
| Wares, D. F. | 2000 | 14 | 3,051 | 2,298 | 0.61 | 75.32 | Low quality |
| Whalen, C. C. | 2011 | 114 | 1,918 | 1,918 | 5.94 | 100.00 | High quality |
| Woldesemayat, E. M. | 2015 | 26 | 21,774 | 21,774 | 0.12 | 100.00 | Low quality |
| Wood, R. | 2007 | 12 | 1,150 | 762 | 1.57 | 66.26 | Low quality |
| Wyk, S. S. van | 2012 | 3 | 12 | 12 | 25.00 | 100.00 | High quality |
| Xavier, T. | 1992 | 72 | 35,000 | 35,000 | 0.21 | 100.00 | Low quality |
| Xavier, T. | 1992 | 41 | 11,808 | 11,808 | 0.35 | 100.00 | Low quality |
| Yared, Tadesse | 2016 | 5 | 282 | 237 | 2.11 | 84.04 | Low quality |
| Yassin, M. A. | 2013 | 69 | 1,290 | 1,290 | 5.35 | 100.00 | Low quality |
| Yimer, S. | 2009 | 38 | 47,478 | 47,478 | 0.08 | 100.00 | Low quality |
| Zachariah, R. | 2003 | 4 | 113 | 113 | 3.54 | 100.00 | Low quality |
| Zachariah, R. | 2003 | 4 | 348 | 348 | 1.15 | 100.00 | Low quality |
| Zaeh, S. | 2013 | 5 | 751 | 738 | 0.68 | 98.27 | High quality |

**REFERENCES**

1. Centers for Disease, C. and Prevention, *Rapid assessment of tuberculosis in a large prison system--Botswana, 2002.* MMWR Morb Mortal Wkly Rep, 2003. **52**(12): p. 250-2.

2. India Tuberculosis-Diabetes Study, G., *Screening of patients with tuberculosis for diabetes mellitus in India.* Trop Med Int Health, 2013. **18**(5): p. 636-45.

3. Abebe, D.S., et al., *Prevalence of pulmonary tuberculosis and associated risk factors in Eastern Ethiopian prisons.* Int J Tuberc Lung Dis, 2011. **15**(5): p. 668-73.

4. Adane, K., et al., *Half of Pulmonary Tuberculosis Cases Were Left Undiagnosed in Prisons of the Tigray Region of Ethiopia: Implications for Tuberculosis Control.* PLoS One, 2016. **11**(2): p. e0149453.

5. Addis, Z., et al., *Prevalence of smear positive pulmonary tuberculosis in Gondar prisoners, North West Ethiopia.* Asian Pac J Trop Med, 2015. **8**(2): p. 127-31.

6. Adelman, M.W., et al., *Intensified tuberculosis case finding among HIV-infected persons using a WHO symptom screen and Xpert((R)) MTB/RIF.* Int J Tuberc Lung Dis, 2015. **19**(10): p. 1197-203.

7. Adetifa, I.M., et al., *A tuberculosis nationwide prevalence survey in Gambia, 2012.* Bull World Health Organ, 2016. **94**(6): p. 433-41.

8. Ahmad Khan, F., et al., *Performance of symptom-based tuberculosis screening among people living with HIV: not as great as hoped.* AIDS, 2014. **28**(10): p. 1463-1472.

9. Akhtar, S., et al., *Hyperendemic pulmonary tuberculosis in peri-urban areas of Karachi, Pakistan.* BMC Public Health, 2007. **7**: p. 70.

10. Alemayehu, M., et al., *Active tuberculosis case finding and detection of drug resistance among HIV-infected patients: A cross-sectional study in a TB endemic area, Gondar, Northwest Ethiopia.* International Journal of Mycobacteriology, 2014. **3**(2): p. 132-138.

11. Ali, N.S., S.F. Hussain, and S.I. Azam, *Is there a value of mantoux test and erythrocyte sedimentation rate in pre-employment screening of health care workers for tuberculosis in a high prevalence country?* Int J Tuberc Lung Dis, 2002. **6**(11): p. 1012-6.

12. Ali, S., et al., *Prevalence of Pulmonary Tuberculosis among Prison Inmates in Ethiopia, a Cross-Sectional Study.* PLoS ONE, 2015. **10**(12).

13. Alonso-Echanove, J., et al., *Occupational transmission of Mycobacterium tuberculosis to health care workers in a university hospital in Lima, Peru.* Clin Infect Dis, 2001. **33**(5): p. 589-96.

14. Aluoch, J.A., et al., *A fourth study of case-finding methods for pulmonary tuberculosis in Kenya.* Trans R Soc Trop Med Hyg, 1982. **76**(5): p. 679-91.

15. Aluoch, J.A., et al., *A study of the use of maternity and child welfare clinics in case-finding for pulmonary tuberculosis in Kenya.* Tubercle, 1987. **68**(2): p. 93-103.

16. Aluoch, J.A., et al., *Studies of case-finding for pulmonary tuberculosis in outpatients at 4 district hospitals in Kenya.* Tubercle, 1985. **66**(4): p. 237-249.

17. Amare, D., et al., *Prevalence of pulmonary TB and spoligotype pattern of Mycobacterium tuberculosis among TB suspects in a rural community in Southwest Ethiopia.* BMC Infectious Diseases, 2012. **12**(54): p. (13 March 2012).

18. Aneja, K.S., N.K. Menon, and A.K. Chakraborty, *Feasibility of involvement of the multi purpose workers in case finding in district tuberculosis programme.* Indian Journal of Tuberculosis, 1980. **27**(4): p. 158-166.

19. Anigilaje, E.A., et al., *Tuberculosis, before and after Antiretroviral Therapy among HIV-Infected Children in Nigeria: What Are the Risk Factors?* PLoS One, 2016. **11**(5): p. e0156177.

20. Arscott-Mills, T., et al., *Yield of screening for TB and HIV among children failing to thrive in Botswana.* J Trop Pediatr, 2014. **60**(1): p. 27-32.

21. Assefa, D., et al., *Intensified tuberculosis case finding among people living with the human immunodeficiency virus in a hospital clinic in Ethiopia.* Int J Tuberc Lung Dis, 2011. **15**(3): p. 411-3.

22. Asuquo, A.E., et al., *A public-private partnership to reduce tuberculosis burden in Akwa Ibom State, Nigeria.* Int J Mycobacteriol, 2015. **4**(2): p. 143-50.

23. Auld, A.F., et al., *Wide Variations in Compliance with Tuberculosis Screening Guidelines and Tuberculosis Incidence between Antiretroviral Therapy Facilities - Cote d'Ivoire.* PLoS One, 2016. **11**(6): p. e0157059.

24. Ayles, H., et al. *Effect of household and community interventions on the burden of tuberculosis in southern Africa: The ZAMSTAR community-randomised trial*. The Lancet, 2013. **382**, 1183-1194.

25. Bakari, M., et al., *Basis for treatment of tuberculosis among HIV-infected patients in Tanzania: the role of chest x-ray and sputum culture.* BMC Infectious Diseases, 2008. **8**(32): p. (6 March 2008).

26. Balasubramanian, R., et al., *Feasibility of involving literate tribal youths in tuberculosis case-finding in a tribal area in Tamil Nadu.* Tubercle and Lung Disease, 1995. **76**(4): p. 355-359.

27. Balcha, T.T., et al., *Intensified tuberculosis case-finding in HIV-positive adults managed at Ethiopian health centers: diagnostic yield of Xpert MTB/RIF compared with smear microscopy and liquid culture.* PLoS One, 2014. **9**(1): p. e85478.

28. Banda, H.T., et al., *Prevalence of smear-positive pulmonary tuberculosis among prisoners in Malawi: a national survey.* Int J Tuberc Lung Dis, 2009. **13**(12): p. 1557-9.

29. Banu, S., et al., *Epidemiology of tuberculosis in an urban slum of Dhaka City, Bangladesh.* PLoS One, 2013. **8**(10): p. e77721.

30. Banu, S., et al., *Effect of active case finding on prevalence and transmission of pulmonary tuberculosis in Dhaka Central Jail, Bangladesh.* PLoS One, 2015. **10**(5): p. e0124976.

31. Baskaran, D., et al., *Prevalence and risk factors for adult pulmonary tuberculosis in a metropolitan city of south India.* PLoS ONE, 2015. **10**(4): p. e0124260.

32. Bassett, I.V., et al., *Sizanani: A Randomized Trial of Health System Navigators to Improve Linkage to HIV and TB Care in South Africa.* J Acquir Immune Defic Syndr, 2016. **73**(2): p. 154-60.

33. Bassett, I.V., et al., *Intensive tuberculosis screening for HIV-infected patients starting antiretroviral therapy in Durban, South Africa.* Clin Infect Dis, 2010. **51**(7): p. 823-9.

34. Becerra, M.C., et al., *Expanding tuberculosis case detection by screening household contacts.* Public Health Rep, 2005. **120**(3): p. 271-7.

35. Beyene, M., et al., *Prevalence of smear positive pulmonary tuberculosis among prisoners in North Gondar Zone Prison, northwest Ethiopia.* BMC Infectious Diseases, 2012. **12**(352): p. (15 December 2012).

36. Beyers, N., et al., *A prospective evaluation of children under the age of 5 years living in the same household as adults with recently diagnosed pulmonary tuberculosis.* International Journal of Tuberculosis and Lung Disease, 1997. **1**(1): p. 38-43.

37. Bhat, P.G., et al., *Intensified tuberculosis case finding among malnourished children in nutritional rehabilitation centres of Karnataka, India: missed opportunities.* PLoS ONE, 2013. **8**(12): p. e84255.

38. Binepal, G., et al., *Screening difficult-to-reach populations for tuberculosis using a mobile medical unit, Punjab India.* Public Health Action, 2015. **5**(4): p. 241-5.

39. Bjerregaard-Andersen, M., et al., *Tuberculosis burden in an urban population: a cross sectional tuberculosis survey from Guinea Bissau.* BMC Infect Dis, 2010. **10**(96): p. 96.

40. Bjerrum, S., et al., *Tuberculosis and non-tuberculous mycobacteria among HIV-infected individuals in Ghana.* Trop Med Int Health, 2016. **21**(9): p. 1181-90.

41. Bloss, E., et al., *Lessons learned during tuberculosis screening in public medical clinics in Francistown, Botswana.* Int J Tuberc Lung Dis, 2012. **16**(8): p. 1030-2.

42. Boon, S.d., et al., *Comparison of symptoms and treatment outcomes between actively and passively detected tuberculosis cases: the additional value of active case finding.* Epidemiology and Infection, 2008. **136**(10): p. 1342-1349.

43. Burmen, B., et al., *Tuberculosis screening outcomes for newly diagnosed persons living with HIV, Nyanza Province, Kenya, 2009.* Int J Tuberc Lung Dis, 2016. **20**(1): p. 79-84.

44. Cadmus, S.I., et al., *Exposure of dentists to Mycobacterium tuberculosis, Ibadan, Nigeria.* Emerg Infect Dis, 2010. **16**(9): p. 1479-81.

45. Cassels, A., et al., *Tuberculosis case-finding in Eastern Nepal.* Tubercle, 1982. **63**(3): p. 175-85.

46. Chamie, G., et al., *Leveraging rapid community-based HIV testing campaigns for non-communicable diseases in rural Uganda.* PLoS One, 2012. **7**(8): p. e43400.

47. Tuberculosis Research Centre, I.C.o.M.R.I., Chennai, India *Risk of tuberculosis among contacts of isoniazid-resistant and isoniazid-susceptible cases*. The international journal of tuberculosis and lung disease : the official journal of the International Union against Tuberculosis and Lung Disease, 2011. **15**, 782-8 DOI: 10.5588/ijtld.09.0327.

48. Chheng, P., et al., *Tuberculosis case finding in first-degree relative contacts not living with index tuberculosis cases in kampala, uganda.* Clinical Epidemiology, 2015. **7**: p. 411-419.

49. Chheng, P., et al., *Pulmonary tuberculosis among patients visiting a voluntary confidential counseling and testing center, Cambodia.* International Journal of Tuberculosis and Lung Disease, 2008. **12**(3 Suppl.1): p. S54-S62.

50. Chisti, M.J., et al., *A prospective study of the prevalence of tuberculosis and bacteraemia in Bangladeshi children with severe malnutrition and pneumonia including an evaluation of Xpert MTB/RIF assay.* PLoS ONE, 2014. **9**(4): p. e93776.

51. Churchyard, G.J., et al., *Twelve-monthly versus six-monthly radiological screening for active case-finding of tuberculosis: A randomised controlled trial.* Thorax, 2011. **66**(2): p. 134-139.

52. Churchyard, G.J., et al., *A trial of mass isoniazid preventive therapy for tuberculosis control.* N Engl J Med, 2014. **370**(4): p. 301-10.

53. Churchyard, G.J., et al., *Symptom and chest radiographic screening for infectious tuberculosis prior to starting isoniazid preventive therapy: yield and proportion missed at screening.* Aids, 2010. **24 Suppl 5**: p. S19-27.

54. Claassens, M., et al., *High prevalence of tuberculosis and insufficient case detection in two communities in the Western Cape, South Africa.* PLoS ONE, 2013. **8**(4): p. e58689.

55. Clarke, M., et al., *DOTS for temporary workers in the agricultural sector. An exploratory study in tuberculosis case detection.* Curationis, 2003. **26**(4): p. 66-71.

56. Colvin, C., et al., *Evaluation of community-based interventions to improve TB case detection in a rural district of Tanzania.* Glob Health Sci Pract, 2014. **2**(2): p. 219-25.

57. Corbett, E.L., et al., *Comparison of two active case-finding strategies for community-based diagnosis of symptomatic smear-positive tuberculosis and control of infectious tuberculosis in Harare, Zimbabwe (DETECTB): a cluster-randomised trial.* Lancet (British edition), 2010. **376**(9748): p. 1244-1253.

58. Corbett, E.L., et al., *Prevalent infectious tuberculosis in Harare, Zimbabwe: burden, risk factors and implications for control.* Int J Tuberc Lung Dis, 2009. **13**(10): p. 1231-7.

59. Corbett, E.L., et al., *Human immunodeficiency virus and the prevalence of undiagnosed tuberculosis in African gold miners.* Am J Respir Crit Care Med, 2004. **170**(6): p. 673-9.

60. Costenaro, P., et al., *Implementation of the WHO 2011 Recommendations for Isoniazid Preventive Therapy (IPT) in Children Living with HIV/AIDS: A Ugandan Experience.* Journal of Acquired Immune Deficiency Syndromes, 2016. **71**(1): p. e1-e8.

61. Creswell, J., et al., *An evaluation of systematic tuberculosis screening at private facilities in Karachi, Pakistan.* PLoS One, 2014. **9**(4): p. e93858.

62. Crook, A., et al. *Tuberculosis incidence is high in HIV-infected African children but is reduced by co-trimoxazole and time on antiretroviral therapy*. BMC medicine, 2016. **14**, 50 DOI: 10.1186/s12916-016-0593-7.

63. Datiko, D.G. and B. Lindtjørn, *Health extension workers improve tuberculosis case detection and treatment success in southern Ethiopia: a community randomized trial.* PLoS ONE, 2009(No.May): p. e5443.

64. Day, J.H., et al., *Screening for tuberculosis prior to isoniazid preventive therapy among HIV-infected gold miners in South Africa.* Int J Tuberc Lung Dis, 2006. **10**(5): p. 523-9.

65. Deery, C.B., et al., *A home tracing program for contacts of people with tuberculosis or HIV and patients lost to care.* International Journal of Tuberculosis and Lung Disease, 2014. **18**(5): p. 534-540+i.

66. Den Boon, S., et al., *An evaluation of symptom and chest radiographic screening in tuberculosis prevalence surveys.* International Journal of Tuberculosis and Lung Disease, 2006. **10**(8): p. 876-882.

67. Dereje, H., et al., *The additional yield of GeneXpert MTB/RIF test in the diagnosis of pulmonary tuberculosis among household contacts of smear positive TB cases.* International Journal of Infectious Diseases, 2016. **49**: p. 179-184.

68. Deribew, A., et al., *Investigation outcomes of tuberculosis suspects in the health centers of Addis Ababa, Ethiopia.* PLoS ONE, 2011(No.April): p. e18614.

69. Dimairo, M., et al., *The risk and timing of tuberculosis diagnosed in smear-negative TB suspects: A 12 month cohort study in Harare, Zimbabwe.* PLoS ONE, 2010. **5**(7).

70. Dorman, S.E., et al., *Performance characteristics of the cepheid Xpert MTB/RIF test in a tuberculosis prevalence survey.* PLoS ONE, 2012. **7**(8): p. e43307.

71. Drain, P.K., et al., *Diagnostic accuracy of a point-of-care urine test for tuberculosis screening among newly-diagnosed HIV-infected adults: a prospective, clinic-based study.* BMC Infect Dis, 2014. **14**: p. 110.

72. Drain, P.K., et al., *Value of urine lipoarabinomannan grade and second test for optimizing clinic-based screening for HIV-associated pulmonary tuberculosis.* J Acquir Immune Defic Syndr, 2015. **68**(3): p. 274-80.

73. Eang, M.T., et al., *Early detection of tuberculosis through community-based active case finding in Cambodia.* BMC Public Health, 2012. **12**(1).

74. Elden, S., et al., *Integrating intesified case finding of tuberculosis into HIV care: an evaluation from rural Swaziland.* BMC Health Services Research, 2011. **11**(118): p. (23 May 2011).

75. Fairall, L.R., et al., *Effect of educational outreach to nurses on tuberculosis case detection and primary care of respiratory illness: pragmatic cluster randomised controlled trial.* British Medical Journal (Clinical Research edition), 2005. **331**(7519): p. 750-754.

76. Fatima, R., et al., *Success of active tuberculosis case detection among high-risk groups in urban slums in Pakistan.* International Journal of Tuberculosis and Lung Disease, 2014. **18**(9): p. 1099-1104.

77. Fox, G.J., et al., *Contact investigation in households of patients with tuberculosis in Hanoi, Vietnam: a prospective cohort study.* PLoS ONE, 2012. **7**(11): p. e49880.

78. Fuge, T.G. and S.Y. Ayanto, *Prevalence of smear positive pulmonary tuberculosis and associated risk factors among prisoners in Hadiya Zone prison, Southern Ethiopia.* BMC Res Notes, 2016. **9**: p. 201.

79. Garcia-Prats, A.J., et al., *Children exposed to multidrug-resistant tuberculosis at a home-based day care centre: a contact investigation.* International Journal of Tuberculosis and Lung Disease, 2014. **18**(11): p. 1292-1298.

80. Gashu, Z., et al., *The Yield of Community-Based "Retrospective" Tuberculosis Contact Investigation in a High Burden Setting in Ethiopia.* PLoS One, 2016. **11**(8): p. e0160514.

81. Gebretsadik, B., et al., *Population-based prevalence survey of tuberculosis in the Tigray region of Ethiopia.* BMC Infectious Diseases, 2013. **13**(448): p. (28 September 2013).

82. Getahun, H. and D. Maher, *Contribution of 'TB clubs' to tuberculosis control in a rural district in Ethiopia.* International Journal of Tuberculosis and Lung Disease, 2000. **4**(2): p. 174-178.

83. Ghiya, R., et al., *Clinico-epidemiological profile of HIV/TB coinfected patients in Vadodara, Gujarat.* Indian Journal of Sexually Transmitted Diseases, 2009. **30**(1): p. 10-15.

84. Giri, P.A., J.D. Deshpande, and D.B. Phalke, *Prevalence of pulmonary tuberculosis among HIV positive patients attending antiretroviral therapy clinic.* North American Journal of Medical Sciences, 2013. **5**(6): p. 367-370.

85. Gounder, C.R., et al., *Diagnostic accuracy of a urine lipoarabinomannan enzyme-linked immunosorbent assay for screening ambulatory HIV-infected persons for tuberculosis.* J Acquir Immune Defic Syndr, 2011. **58**(2): p. 219-23.

86. Gounder, C.R., et al., *Active tuberculosis case-finding among pregnant women presenting to antenatal clinics in Soweto, South Africa.* JAIDS, Journal of Acquired Immune Deficiency Syndromes, 2011. **57**(4): p. e77-e84.

87. Govindasamy, D., et al., *Linkage to HIV, TB and non-communicable disease care from a mobile testing unit in Cape Town, South Africa.* PLoS One, 2013. **8**(11): p. e80017.

88. Gupta, A., et al., *Active case finding for tuberculosis among people who inject drugs on methadone treatment in Dar es Salaam, Tanzania.* International Journal of Tuberculosis and Lung Disease, 2014. **18**(7): p. 793-798.

89. Gupta, A., et al. *Postpartum tuberculosis incidence and mortality among HIV-infected women and their infants in Pune, India, 2002-2005*. Clinical infectious diseases : an official publication of the Infectious Diseases Society of America, 2007. **45**, 241-9 DOI: 10.1086/518974.

90. Gyawali, N., et al., *Prevalence of tuberculosis in household contacts of sputum smears positive cases and associated demographic risk factors.* Nepal Med Coll J, 2012. **14**(4): p. 303-7.

91. Habeenzu, C., et al., *Tuberculosis and multidrug resistance in Zambian prisons, 2000-2001.* Int J Tuberc Lung Dis, 2007. **11**(11): p. 1216-20.

92. Hamusse, S.D., et al., *Primary and secondary anti-tuberculosis drug resistance in Hitossa District of Arsi Zone, Oromia Regional State, Central Ethiopia.* BMC Public Health, 2016. **16**: p. 593.

93. Hanifa, Y., et al., *Tuberculosis among adults starting antiretroviral therapy in South Africa: the need for routine case finding.* Int J Tuberc Lung Dis, 2012. **16**(9): p. 1252-9.

94. Hanifa, Y., et al., *Diagnostic Accuracy of Lateral Flow Urine LAM Assay for TB Screening of Adults with Advanced Immunosuppression Attending Routine HIV Care in South Africa.* PLoS One, 2016. **11**(6): p. e0156866.

95. Harper, I., R. Fryatt, and A. White, *Tuberculosis case finding in remote mountainous areas - Are microscopy camps of any value? Experience from Nepal.* Tubercle and Lung Disease, 1996. **77**(4): p. 384-388.

96. Harris, J.B., et al., *Derivation of a tuberculosis screening rule for sub-Saharan African prisons.* Int J Tuberc Lung Dis, 2014. **18**(7): p. 774-80.

97. Henostroza, G., et al., *High prevalence of tuberculosis in newly enrolled HIV patients in Zambia: need for enhanced screening approach.* Int J Tuberc Lung Dis, 2016. **20**(8): p. 1033-9.

98. Hermans, S., et al., *Implementation and effect of intensified case finding on diagnosis of tuberculosis in a large urban HIV clinic in Uganda: a retrospective cohort study.* BMC Public Health, 2012. **12**: p. 674.

99. Ho, J., et al., *Reassessment of the positive predictive value and specificity of Xpert MTB/RIF: a diagnostic accuracy study in the context of community-wide screening for tuberculosis.* Lancet Infect Dis, 2016.

100. Hoffmann, C.J., et al., *High Prevalence of Pulmonary Tuberculosis but Low Sensitivity of Symptom Screening among HIV-Infected Pregnant Women in South Africa.* PLoS ONE, 2013. **8**(4).

101. van't Hoog, A.H., et al., *High prevalence of pulmonary tuberculosis and inadequate case finding in rural western Kenya.* Am J Respir Crit Care Med, 2011. **183**(9): p. 1245-53.

102. Houlihan, C.F., et al., *The tuberculosis challenge in a rural South African HIV programme.* BMC Infectious Diseases, 2010. **10**(23): p. (10 February 2010).

103. Iroezindu, M.O., et al., *Factors Associated with Prevalent Tuberculosis Among Patients Receiving Highly Active Antiretroviral Therapy in a Nigerian Tertiary Hospital.* Ann Med Health Sci Res, 2016. **6**(2): p. 120-8.

104. Jackson-Sillah, D., et al., *Screening for tuberculosis among 2381 household contacts of sputum-smear-positive cases in The Gambia.* Transactions of the Royal Society of Tropical Medicine and Hygiene, 2007. **101**(6): p. 594-601.

105. Jaganath, D., et al., *Contact investigation for active tuberculosis among child contacts in Uganda.* Clinical Infectious Diseases, 2013. **57**(12): p. 1685-1692.

106. Javaid, A., et al., *Screening outcomes of household contacts of multidrug-resistant tuberculosis patients in Peshawar, Pakistan.* Asian Pac J Trop Med, 2016. **9**(9): p. 909-12.

107. Jerene, D., et al., *The yield of a tuberculosis household contact investigation in two regions of Ethiopia.* International Journal of Tuberculosis and Lung Disease, 2015. **19**(8): p. 898-903.

108. John, S., et al., *Tuberculosis among nomads in Adamawa, Nigeria: outcomes from two years of active case finding.* Int J Tuberc Lung Dis, 2015. **19**(4): p. 463-8.

109. Jones-Lopez, E.C., et al., *Cough Aerosols of Mycobacterium tuberculosis in the Prediction of Incident Tuberculosis Disease in Household Contacts.* Clin Infect Dis, 2016. **63**(1): p. 10-20.

110. Joshi, B., et al., *Impact of intensified case-finding strategies on childhood TB case registration in Nepal.* Public Health Action, 2015. **5**(2): p. 93-8.

111. Joshi, Y.P., P.N. Mishra, and D.D. Joshi, *Prevalence of pulmonary tuberculosis in far Western Nepal.* JNMA J Nepal Med Assoc, 2005. **44**(158): p. 47-50.

112. Kali, P.B., et al., *Combining PMTCT with active case finding for tuberculosis.* J Acquir Immune Defic Syndr, 2006. **42**(3): p. 379-81.

113. Kassim, S., et al., *Tuberculin skin testing to assess the occupational risk of Mycobacterium tuberculosis infection among health care workers in Abidjan, Cote d'Ivoire.* Int J Tuberc Lung Dis, 2000. **4**(4): p. 321-6.

114. Khan, A.J., et al., *Engaging the private sector to increase tuberculosis case detection: an impact evaluation study.* Lancet Infectious Diseases, 2012. **12**(8): p. 608-616.

115. Khanal, S., et al., *Yield of intensified tuberculosis case-finding activities using Xpert® MTB/RIF among risk groups in Nepal.* Public Health Action, 2016. **6**(2): p. 136-141.

116. Khaparde, K., et al., *Evaluation of TB Case Finding through Systematic Contact Investigation, Chhattisgarh, India.* Tuberc Res Treat, 2015. **2015**: p. 670167.

117. Kimerling, M.E., et al., *Prevalence of pulmonary tuberculosis among HIV-infected persons in a home care program in Phnom Penh, Cambodia.* International Journal of Tuberculosis and Lung Disease, 2002. **6**(11): p. 988-994.

118. Kliner, M., et al., *Development and testing of models of tuberculosis contact tracing in rural southern Africa.* Public Health Action, 2013. **3**(4): p. 299-303.

119. Koenig, S.P., et al., *Tuberculosis in the aftermath of the 2010 earthquake in Haiti.* Bull World Health Organ, 2015. **93**(7): p. 498-502.

120. Kranzer, K., et al., *Feasibility, yield, and cost of active tuberculosis case finding linked to a mobile HIV service in Cape Town, South Africa: a cross-sectional study.* PLoS Medicine, 2012. **9**(8): p. e1001281.

121. Kufa, T., et al., *The incidence of tuberculosis among hiv-positive individuals with high CD4 counts: implications for policy.* BMC Infect Dis, 2016. **16**: p. 266.

122. Kufa, T., et al., *Undiagnosed tuberculosis among HIV clinic attendees: Association with antiretroviral therapy and implications for intensified case finding, isoniazid preventive therapy, and infection control.* Journal of Acquired Immune Deficiency Syndromes, 2012. **60**(2): p. e22-e28.

123. Kwabla, M.P., D.K. Ameme, and P. Nortey, *Pulmonary Tuberculosis and Its Risk Factors among Inmates of a Ghanaian Prison.* 2015.

124. LaCourse, S.M., et al., *Use of xpert for the diagnosis of pulmonary tuberculosis in severely malnourished hospitalized Malawian children.* Pediatric Infectious Disease Journal, 2014. **33**(11): p. 1200-1202.

125. LaCourse, S.M., et al., *Tuberculosis case finding in HIV-infected pregnant women in Kenya reveals poor performance of symptom screening and rapid diagnostic tests.* JAIDS, Journal of Acquired Immune Deficiency Syndromes, 2016. **71**(2): p. 219-227.

126. Lala, S.G., et al., *Integrated Source Case Investigation for Tuberculosis (TB) and HIV in the Caregivers and Household Contacts of Hospitalised Young Children Diagnosed with TB in South Africa: An Observational Study.* PLoS One, 2015. **10**(9): p. e0137518.

127. Lawn, S.D., et al. *Screening for HIV-associated tuberculosis and rifampicin resistance before antiretroviral therapy using the Xpert MTB/RIF assay: a prospective study*. PLoS medicine, 2011. **8**, e1001067.

128. Lawn, S.D., et al., *Tuberculosis during the first year of antiretroviral therapy in a South African cohort using an intensive pretreatment screening strategy.* AIDS, 2010. **24**(9): p. 1323-1328.

129. Lebina, L., et al., *The Use of Xpert MTB/Rif for Active Case Finding among TB Contacts in North West Province, South Africa.* Tuberc Res Treat, 2016. **2016**: p. 4282313.

130. Lewis, J.J., et al., *HIV infection does not affect active case finding of tuberculosis in South African gold miners.* American Journal of Respiratory and Critical Care Medicine, 2009. **180**(12): p. 1271-1278.

131. Liaquat, A., et al., *Concomitant presence of culture-proven active pulmonary tuberculosis in patients with chronic obstructive pulmonary disease - A hospital based study.* Pak J Med Sci, 2015. **31**(6): p. 1344-8.

132. Lorent, N., et al., *Is frontloaded sputum microscopy an option in active tuberculosis case finding?* International Journal of Tuberculosis and Lung Disease, 2015. **19**(1): p. 91-96.

133. Lorent, N., et al., *Challenges from tuberculosis diagnosis to care in community-based active case finding among the urban poor in Cambodia: A mixed-methods study.* PLoS ONE, 2015. **10**(7).

134. Mabuto, T., et al., *Tuberculosis active case finding: uptake and diagnostic yield among minibus drivers in urban South Africa.* BMC Public Health, 2015. **15**: p. 242.

135. Madhavi, P., et al., *Tuberculosis contact screening and isoniazid preventive therapy in a South Indian District: operational issues for programmatic consideration.* PLoS ONE, 2011(No.July): p. e22500.

136. Majumder, A., et al., *Screening for active tuberculosis in a diabetes mellitus clinic in Soweto, South Africa.* Int J Tuberc Lung Dis, 2016. **20**(7): p. 992-3.

137. Mallikarjun, V.J., et al., *Bidirectional Screening of Tuberculosis Patients for Diabetes Mellitus and Diabetes Patients for Tuberculosis.* Diabetes Metabolism Journal. **37**(4): p. 291-295.

138. Manzoor, S., Z. Tahir, and A. Anjum, *Prevalence of HIV and tuberculosis among jail inmates in Lahore - Pakistan.* Biomedica, 2009. **25**(Jan.-Jun.): p. 36-41.

139. Maritz, E.R., et al., *Source case identification in HIV-exposed infants and tuberculosis diagnosis in an isoniazid prevention study.* Int J Tuberc Lung Dis, 2016. **20**(8): p. 1060-4.

140. Martinez, L., et al., *Infectiousness of HIV Seropositive Tuberculosis Patients in a High-burden African Setting.* Am J Respir Crit Care Med, 2016.

141. Matee, M., et al., *Sputum microscopy for the diagnosis of HIV-associated pulmonary tuberculosis in Tanzania.* BMC Public Health, 2008. **8**(68): p. (21 February 2008).

142. McDowell, M., et al., *Expanding tuberculosis case notification among marginalized groups in Bangladesh through peer sputum collection.* Public Health Action, 2015. **5**(2): p. 119-21.

143. Moyo, S., et al. *Tuberculosis case finding for vaccine trials in young children in high-incidence settings: A randomised trial*. International Journal of Tuberculosis and Lung Disease, 2012. **16**, 185-191 DOI: <http://dx.doi.org/10.5588/ijtld.11.0348>.

144. Mridul, G., A.A. Saibannavar, and K. Vinod, *Household symptomatic contact screening of newly diagnosed sputum smears positive tuberculosis patients - an effective case detection tool.* Lung India, 2016. **33**(2): p. 159-162.

145. Mtwangambate, G., et al., *'Cough-triggered' tuberculosis screening among adults with diabetes in Tanzania.* Diabet Med, 2014. **31**(5): p. 600-5.

146. Mugisha, B., et al., *Tuberculosis case finding and preventive therapy in an HIV voluntary counseling and testing center in Uganda.* Int J Tuberc Lung Dis, 2006. **10**(7): p. 761-7.

147. Mulenga, H., et al. *The Role of Clinical Symptoms in the Diagnosis of Intrathoracic Tuberculosis in Young Children*. The Pediatric infectious disease journal, 2015. **34**, 1157-62 DOI: 10.1097/INF.0000000000000847.

148. Mupfumi, L., et al. *Impact of Xpert MTB/RIF on antiretroviral therapy-associated tuberculosis and mortality: A pragmatic randomized controlled trial*. Open Forum Infectious Diseases, 2014. **1**, DOI: 10.1093/ofid/ofu038.

149. Musa, B.M., et al., *Incidence of tuberculosis and immunological profile of TB/HIV co-infected patients in Nigeria.* Ann Thorac Med, 2015. **10**(3): p. 185-92.

150. Mushtaque, A., et al., *Control of tuberculosis by community health workers in Bangladesh.* Lancet (British edition), 1997. **350**(9072): p. 169-172.

151. Nachega, J., et al., *Tuberculosis active case-finding in a mother-to-child HIV transmission prevention programme in Soweto, South Africa.* AIDS, 2003. **17**(9): p. 1398-1400.

152. Nair, A., et al., *Prevalence of pulmonary tuberculosis in young adult patients with Type 1 diabetes mellitus in India.* Multidiscip Respir Med, 2016. **11**: p. 22.

153. Nair, D., et al., *Household Contact Screening and Yield of Tuberculosis Cases-A Clinic Based Study in Chennai, South India.* PLoS One, 2016. **11**(9): p. e0162090.

154. Nakanjako, D., et al., *Tuberculosis and human immunodeficiency virus co-infections and their predictors at a hospital-based HIV/AIDS clinic in Uganda.* Int J Tuberc Lung Dis, 2010. **14**(12): p. 1621-8.

155. Narang, P., et al., *Smear and culture positive cases of pulmonary tuberculosis found among symptomatics surveyed in Wardha district.* Indian Journal of Tuberculosis, 1992. **39**(3): p. 159-163.

156. Nduba, V., et al., *Prevalence of tuberculosis in adolescents, western Kenya: implications for control programs.* International Journal of Infectious Diseases, 2015. **35**: p. 11-17.

157. Ndwiga, C., et al., *Feasibility and effect of integrating tuberculosis screening and detection in postnatal care services: an operations research study.* BMC Health Serv Res, 2013. **13**: p. 99.

158. Ngadaya, E.S., et al., *Detection of pulmonary tuberculosis among patients with cough attending outpatient departments in Dar Es Salaam, Tanzania: does duration of cough matter?* BMC Health Serv Res, 2009. **9**: p. 112.

159. Nguyen, D.T., et al., *Yield of chest radiograph in tuberculosis screening for HIV-infected persons at a district-level HIV clinic.* Int J Tuberc Lung Dis, 2016. **20**(2): p. 211-7.

160. Nliwasa, M., et al., *The Sensitivity and Specificity of Loop-Mediated Isothermal Amplification (LAMP) Assay for Tuberculosis Diagnosis in Adults with Chronic Cough in Malawi.* PLoS One, 2016. **11**(5): p. e0155101.

161. Noeske, J., et al., *Pulmonary tuberculosis in the Central Prison of Douala, Cameroon.* East Afr Med J, 2006. **83**(1): p. 25-30.

162. Noeske, J., N. Ndi, and S. Mbondi, *Controlling tuberculosis in prisons against confinement conditions: a lost case? Experience from Cameroon.* Int J Tuberc Lung Dis, 2011. **15**(2): p. 223-7, i.

163. Nsanzumuhire, H., et al., *A third study of case-finding methods for pulmonary tuberculosis in Kenya, including the use of community leaders.* Tubercle, 1981. **62**(2): p. 79-94.

164. Ntinginya, E.N., et al., *Performance of the Xpert® MTB/RIF assay in an active case-finding strategy: a pilot study from Tanzania.* International Journal of Tuberculosis and Lung Disease, 2012. **16**(11): p. 1468-1470.

165. Nyangulu, D.S., et al., *Tuberculosis in a prison population in Malawi.* Lancet, 1997. **350**(9087): p. 1284-7.

166. Ogbudebe, C.L., et al., *Reaching the underserved: Active tuberculosis case finding in urban slums in southeastern Nigeria.* Int J Mycobacteriol, 2015. **4**(1): p. 18-24.

167. Okada, K., et al., *Epidemiological impact of mass tuberculosis screening: A 2-year follow-up after a national prevalence survey.* International Journal of Tuberculosis and Lung Disease, 2012. **16**(12): p. 1619-1624.

168. Olofin, I.O., et al., *Active tuberculosis in HIV-exposed Tanzanian children up to 2 years of age: early-life nutrition, multivitamin supplementation and other potential risk factors.* Journal of Tropical Pediatrics, 2016. **62**(1): p. 29-37.

169. Otero, L., et al., *A prospective longitudinal study of tuberculosis among household contacts of smear-positive tuberculosis cases in Lima, Peru.* BMC Infect Dis, 2016. **16**: p. 259.

170. Page-Shipp, L., et al., *Successes, challenges and lessons from a novel deployment of Xpert® MTB/RIF at a major South African public event.* International Journal of Tuberculosis and Lung Disease, 2014. **18**(4): p. 438-440.

171. Pathak, R.R., et al., *Can Intensified Tuberculosis Case Finding Efforts at Nutrition Rehabilitation Centers Lead to Pediatric Case Detection in Bihar, India?* J Tuberc Res, 2016. **4**(1): p. 46-54.

172. Prasad, B.M., et al., *Experience of active tuberculosis case finding in nearly 5 million households in India.* Public Health Action, 2016. **6**(1): p. 15-8.

173. Pronyk, P.M., et al., *Active case finding: understanding the burden of tuberculosis in rural South Africa.* International Journal of Tuberculosis and Lung Disease, 2001. **5**(7): p. 611-618.

174. Puryear, S., et al., *Yield of contact tracing from pediatric tuberculosis index cases in Gaborone, Botswana.* Int J Tuberc Lung Dis, 2013. **17**(8): p. 1049-55.

175. Rangaka, M.X., et al., *Effect of antiretroviral therapy on the diagnostic accuracy of symptom screening for intensified tuberculosis case finding in a South African HIV clinic.* Clinical Infectious Diseases, 2012. **55**(12): p. 1698-1706.

176. Ray, D. and R. Abel, *Incidence of smear-positive pulmonary tuberculosis from 1981-83 in a rural area under an active health care programme in South India.* Tubercle and Lung Disease, 1995. **76**(3): p. 190-195.

177. Reepalu, A., et al., *Factors associated with early mortality in HIV-positive men and women investigated for tuberculosis at Ethiopian health centers.* PLoS ONE, 2016. **11**(6): p. e0156602.

178. Rekha Devi, K., et al., *Active detection of tuberculosis and paragonimiasis in the remote areas in North-Eastern India using cough as a simple indicator.* Pathog Glob Health, 2013. **107**(3): p. 153-6.

179. Roy, M., et al., *Use of symptom screening and sputum microscopy testing for active tuberculosis case detection among HIV-infected patients in real-world clinical practice in Uganda.* Journal of Acquired Immune Deficiency Syndromes, 2016. **72**(5): p. e86-e91.

180. Salazar-Vergara, R.M.L., et al., *Tuberculosis infection and disease in children living in households of Filipino patients with tuberculosis: A preliminary report.* International Journal of Tuberculosis and Lung Disease, 2003. **7**(12 SUPPL. 3): p. S494-S500.

181. Santha, T., et al., *Are community surveys to detect tuberculosis in high prevalence areas useful? Results of a comparative study from Tiruvallur District, South India.* International Journal of Tuberculosis and Lung Disease, 2003. **7**(3): p. 258-265.

182. Schalkwyk, C.v., et al., *Incidence of TB and HIV in prospectively followed household contacts of TB index patients in South Africa.* PLoS ONE, 2014. **9**(4): p. e95372.

183. Seddon, J.A., et al., *Risk factors for infection and disease in child contacts of multidrug-resistant tuberculosis: a cross-sectional study.* BMC Infectious Diseases, 2013. **13**(392): p. (26 August 2013).

184. Sekandi, J.N., et al., *Yield of undetected tuberculosis and human immunodeficiency virus coinfection from active case finding in urban Uganda.* Int J Tuberc Lung Dis, 2014. **18**(1): p. 13-9.

185. Sekandi, J.N., et al., *Active case finding of undetected tuberculosis among chronic coughers in a slum setting in Kampala, Uganda.* Int J Tuberc Lung Dis, 2009. **13**(4): p. 508-13.

186. Shabbir, I., R. Iqbal, and E. Qadeer, *Screening of tuberculosis in Kharal Abbasian Azad Kashmir.* Pak. j. med. res, 2009. **48**(1): p. 1-3.

187. Shah, S.A., et al., *Active contact investigation and treatment support: an integrated approach in rural and urban Sindh, Pakistan.* Int J Tuberc Lung Dis, 2013. **17**(12): p. 1569-74.

188. Shah, S., et al., *Intensified tuberculosis case finding among HIV-infected persons from a voluntary counseling and testing center in addis ababa, ethiopia.* Journal of Acquired Immune Deficiency Syndromes, 2009. **50**(5): p. 537-545.

189. Shapiro, A.E., et al., *Community-based targeted case finding for tuberculosis and HIV in household contacts of patients with tuberculosis in South Africa.* Am J Respir Crit Care Med, 2012. **185**(10): p. 1110-6.

190. Shargie, E., O. Mørkve, and B. Lindtjørn *Tuberculosis case-finding through a village outreach programme in a rural setting in southern Ethiopia: community randomized trial*. Bulletin of the World Health Organization, 2006. **84**, 112-9 DOI: /S0042-96862006000200011.

191. Sharma, S.K., et al., *Prevalence of tuberculosis in Faridabad district, Haryana State, India.* Indian Journal of Medical Research, 2015. **141**(2): p. 228-235.

192. Shayo, G.A., et al., *Symptom-based screening tool in ruling out active tuberculosis among HIV-infected patients eligible for isoniazid preventive therapy in Tanzania.* Tropical Medicine and International Health, 2014. **19**(6): p. 726-733.

193. Shrivastava, S.R. and P.S. Shrivastava, *Tuberculosis: Active case finding survey in an urban area of India, in 2012.* Journal of Research in Health Sciences, 2013. **13**(1): p. 19-23.

194. Sia, I.G., et al., *Tuberculosis attributed to household contacts in the Philippines.* Int J Tuberc Lung Dis, 2010. **14**(1): p. 122-5.

195. Sinfield, R., et al., *Risk factors for TB infection and disease in young childhood contacts in Malawi.* Annals of Tropical Paediatrics, 2006. **26**(3): p. 205-213.

196. Sretrirutchai, S., et al., *Tuberculosis in Thai prisons: magnitude, transmission and drug susceptibility.* Int J Tuberc Lung Dis, 2002. **6**(3): p. 208-14.

197. Srichand, B., et al., *Childhood tuberculosis in household contacts of newly diagnosed TB patients.* PLoS ONE, 2012. **7**(7): p. e40880.

198. Ssemmondo, E., et al., *Population-based active TB case finding during large-scale mobile HIV testing campaigns in rural Uganda.* J Acquir Immune Defic Syndr, 2016.

199. Suresh, S., et al., *TB-HIV co-infection among pregnant women in Karnataka, South India: A case series.* J Infect Public Health, 2016. **9**(4): p. 465-70.

200. Swindells, S., et al. *Screening for pulmonary tuberculosis in HIV-infected individuals: AIDS Clinical Trials Group Protocol A5253*. International journal of tuberculosis and lung disease, 2013. **17**, 532-9 DOI: 10.5588/ijtld.12.0737.

201. Szkwarko, D., et al., *Implementing intensified tuberculosis case-finding among street-connected youth and young adults in Kenya.* Public Health Action, 2016. **6**(2): p. 142-6.

202. Tadesse, T., et al., *Two-thirds of smear-positive tuberculosis cases in the community were undiagnosed in Northwest Ethiopia: Population based cross-sectional study.* PLoS ONE, 2011. **6**(12).

203. Tadesse, T., et al., *Incidence of smear-positive tuberculosis in Dabat, northern Ethiopia.* International Journal of Tuberculosis and Lung Disease, 2013. **17**(5): p. 630-635.

204. Tamhane, A., et al., *Predictors of smear-negative pulmonary tuberculosis in HIV-infected patients, Battambang, Cambodia.* International Journal of Tuberculosis and Lung Disease, 2009. **13**(3): p. 347-354.

205. Telisinghe, L., et al., *High tuberculosis prevalence in a South African prison: the need for routine tuberculosis screening.* PLoS One, 2014. **9**(1): p. e87262.

206. Thind, D., et al., *An evaluation of 'Ribolola': A household tuberculosis contact tracing programme in North West Province, South Africa.* International Journal of Tuberculosis and Lung Disease, 2012. **16**(12): p. 1643-1648.

207. Tiam, A., et al., *Preventing tuberculosis among HIV-infected pregnant women in Lesotho: the case for rolling out active case finding and isoniazid preventive therapy.* JAIDS, Journal of Acquired Immune Deficiency Syndromes, 2014. **67**(1): p. e5-e11.

208. Topley, J.M., D. Maher, and L.N. Mbewe, *Transmission of tuberculosis to contacts of sputum positive adults in Malawi.* Archives of Disease in Childhood, 1996. **74**(2): p. 140-143.

209. Triasih, R., et al., *Risk of infection and disease with Mycobacterium tuberculosis among children identified through prospective community-based contact screening in Indonesia.* Tropical Medicine and International Health, 2015. **20**(6): p. 737-743.

210. Trinh, T.T., et al., *Implementation and evaluation of an isoniazid preventive therapy pilot program among hiv-infected patients in vietnam, 2008-2010.* Transactions of the Royal Society of Tropical Medicine and Hygiene, 2015. **109**(10): p. 653-659.

211. Tupasi, T.E., et al., *Bacillary disease and health seeking behavior among Filipinos with symptoms of tuberculosis: Implications for control.* International Journal of Tuberculosis and Lung Disease, 2000. **4**(12): p. 1126-1132.

212. Vree, M., et al., *Low tuberculosis notification in mountainous Vietnam is not due to low case detection: a cross-sectional survey.* BMC Infect Dis, 2007. **7**: p. 109.

213. Waako, J., et al., *Burden of tuberculosis disease among adolescents in a rural cohort in Eastern Uganda.* BMC Infectious Diseases, 2013. **13**(349): p. (26 July 2013).

214. Wares, D.F., et al., *Is TB contact screening relevant in a developing country setting? Experiences from eastern Nepal, 1996-1998.* International Journal of Tuberculosis and Lung Disease, 2000. **4**(10): p. 920-924.

215. Whalen, C.C., et al., *Secondary attack rate of tuberculosis in urban households in Kampala, Uganda.* PLoS ONE, 2011(No.February): p. e16137.

216. Woldesemayat, E.M., D.G. Datiko, and L. Bernt, *Follow-up of chronic coughers improves tuberculosis case finding: results from a community-based cohort study in southern Ethiopia.* PLoS ONE, 2015. **10**(2): p. e0116324.

217. Wood, R., et al., *Undiagnosed tuberculosis in a community with high HIV prevalence: Implications for tuberculosis control.* American Journal of Respiratory and Critical Care Medicine, 2007. **175**(1): p. 87-93.

218. Wyk, S.S.v., et al., *Tuberculosis contact investigation in a high-burden setting: house or household?* International Journal of Tuberculosis and Lung Disease, 2012. **16**(2): p. 157-162.

219. Xavier, T., *Strategies to improve case-finding in tuberculosis programme.* Indian Journal of Tuberculosis, 1992. **39**(2): p. 125-126.

220. Yared, T., et al., *Uptake of isoniazid preventive therapy among under-five children: TB contact investigation as an entry point.* PLoS ONE, 2016. **11**(5): p. e0155525.

221. Yassin, M.A., et al., *Innovative Community-Based Approaches Doubled Tuberculosis Case Notification and Improve Treatment Outcome in Southern Ethiopia.* PLoS ONE, 2013. **8**(5).

222. Yimer, S., et al., *Evaluating an active case-finding strategy to identify smear-positive tuberculosis in rural Ethiopia.* Int J Tuberc Lung Dis, 2009. **13**(11): p. 1399-404.

223. Zachariah, R., et al., *Passive versus active tuberculosis case finding and isoniazid preventive therapy among household contacts in a rural district of Malawi.* International Journal of Tuberculosis and Lung Disease, 2003. **7**(11): p. 1033-1039.

224. Zaeh, S., et al., *Improving tuberculosis screening and isoniazid preventive therapy in an HIV clinic in Addis Ababa, Ethiopia.* Int J Tuberc Lung Dis, 2013. **17**(11): p. 1396-401.
