# Supplementary material for: Yield and Coverage of Active Case Finding Interventions for Tuberculosis Control:A Systematic Review and Meta-analysis
Source: Tuberc Res Treat. 2022 Jun 30;2022:9947068. doi: 10.1155/2022/9947068 (PMC9274229; doi:10.1155/2022/9947068)
Supplement: Supplementary 3 — Supplemental Material 3: Table showing Pooled Estimates of Screening Coverage; Based on 256 populations with Data on Numerator and Denominator. [file 9947068.f3.docx]

**Supplemental Table 3: Pooled Estimates of Screening Coverage, Based on 256 Populations with Data on Numerator and Denominator**

| **Characteristics** | **Number Targeted** | **Number Screened** | **Pooled Coverage**  **Estimate (95% CI)** |
| --- | --- | --- | --- |
| Overall | 25,364,350 | 24,997,979 | 93.52 (92.20 – 94.73) |
| Study Design  RCT  Quasi RCT  Prospective  Cross-sectional  Survey  Retrospective chart review | 479,641  123,790  923,049  22,131,792  1,669,515  36,563 | 452,053  64,463  804,220  22,042,788  1,602,683  31,772 | 93.77 (87.84 – 97.82)  52.02 (49.68 – 54.36)  93.00 (87.59 – 96.97)  94.48 (93.21 – 95.62)  94.39 (90.56 – 97.27)  92.12 (78.86 – 99.20) |
| WHO region  Africa  Southeast Asia  Eastern Mediterranean  Western Pacific  The Americas  Multi-regional | 1,840,905  22,910,800  582,012  17,956  11,861  816 | 1,632,261  22,769,888  568,766  15,373  10,984  707 | 91.93 (89.00 – 94.44)  97.06 (95.51 – 98.30)  98.75 (96.30 – 99.95)  79.15 (65.77 – 89.91)  94.80 (79.90 – 100.0)  86.64 (84.14 – 88.81) |
| Recruitment setting  Community  Hospital or clinic  Prisons or residential facility  Workplaces | 24,190,099  893,884  123,128  157,239 | 23,922,235  820,941  116,549  138,254 | 94.72 (93.02 – 96.19)  91.09 (87.67 – 94.01)  96.38 (90.32 – 99.61)  91.69 (85.32 – 96.37) |
| Type of population screened  Contacts  PLWH  General population  High risk for TB exposure^a^  High risk for active TB^b^ | 753,253  87,531  24,185,069  135,272  203,225 | 695,395  79,251  23,932,160  125,556  165,617 | 93.08 (87.78 – 96.98)  92.89 (89.11 – 95.92)  95.31 (93.41 – 96.90)  92.02 (84.53 – 97.20)  91.47 (84.47 – 96.54) |
| Age  Children only  Adults only  Adults and children | 168,501  22,115,772  3,080,077 | 129,871  21,938,552  2,929,556 | 90.47 (84.90 – 94.90)  94.24 (92.59 – 95.70)  92.93 (89.34 – 95.83) |
| Year of publication  2011 to 2016  2000 to 2010  1980 to 1999 | 23,361,962  724,613  1,277,775 | 23,150,738  702,550  1,144,691 | 93.94 (92.45 – 95.27)  93.26 (90.81 – 95.35)  90.89 (81.57 – 97.17) |
| Study quality rating  High quality  Moderate quality  Low quality | 2,783,417  442,706  22,138,227 | 2,624,285  349,126  22,024,568 | 95.75 (94.30 – 96.99)  89.45 (76.13 – 97.75)  89.33 (86.75 – 91.66) |
| Screening modality  Symptom screening  Lab screening^c^  CXR  TST | 24,440,448  300,169  623,295  438 | 24,178,014  224,133  595,514  318 | 94.22 (92.71 – 95.56)  91.01 (85.99 – 95.02)  94.50 (91.21 – 97.06)  72.60 (68.24 – 76.57) |
| Diagnostic modality  Culture &/or GeneXpert MTB/RIF  Microscopy  CXR | 2,688,647  20,884,289  1,791,414 | 2,388,990  20,860,611  1,748,378 | 91.34 (88.79– 93.60)  98.35 (97.45 – 99.07)  92.47 (89.82 – 94.76) |
| Combined Screening and Diagnostic Algorithm  Symptom – Microscopy  Symptom – CXR  Symptom – Culture/Xpert  CXR – Culture/Xpert  Lab^c^ – Culture/Xpert  Other^d^ | 20,843,646  1,744,014  1,852,788  566,465  268,956  88,481 | 20,822,406  1,701,883  1,653,725  541,016  193,931  85,018 | 98.40 (97.46 – 99.13)  93.76 (91.07 – 96.01)  90.20 (86.08 – 93.67)  95.80 (92.38 – 98.25)  90.19 (85.05 – 94.35)  91.26 (84.71 – 96.12) |
| Diagnosis using GeneXpert MTB/RIF  GeneXpert MTB/RIF not used  GeneXpert MTB/RIF used | 24,829,205  535,145 | 24,516,889  481,090 | 93.53 (92.16 – 94.78)  93.46 (87.39 -97.67) |

CI = confidence interval, CXR = chest x-ray, PLWH = people living with HIV infection, RCT = randomized controlled trial, TST = tuberculin skin test

^a^ High risk for TB exposure: health care worker, prisoner, refugee.

^b^ High risk for active TB: diabetes mellitus, pregnancy, miners.

^c^ Includes initial screening using AFB smear (94%), culture (71%), or GeneXpert MTB/RIF (8%)

^d^ Other = microscopy for screening and diagnosis (5), CXR for screening and diagnosis (5), CXR for screening then microscopy for diagnosis (2), TST for screening then culture for diagnosis (1), and microscopy for screening then CXR for diagnosis (2)
