# Supplementary material for: Yield and Coverage of Active Case Finding Interventions for Tuberculosis Control:A Systematic Review and Meta-analysis
Source: Tuberc Res Treat. 2022 Jun 30;2022:9947068. doi: 10.1155/2022/9947068 (PMC9274229; doi:10.1155/2022/9947068)
Supplement: Supplementary 4 — Supplemental Material 4: Table showing Meta-regression of Predictors of Coverage of Active TB Cases following Active Case Finding Activities for 256 Unique Populations. [file 9947068.f4.docx]

**Supplemental Material 4: Meta-regression of Predictors of Coverage of Active TB Cases following Active Case Finding Activities for 256 Unique Populations**

| **Predictors of Coverage** | **Bivariable** |  | **Multivariable Model 1 Screening and Diagnosis Separate** |  | **Multivariable Model 2 Combined Approach** |  | **Multivariable Model 3 Diagnosis by GeneXpert** |  |
| --- | --- | --- | --- | --- | --- | --- | --- | --- |
|  | Beta (95%CI) | P value | Beta (95%CI) | P value | Beta (95%CI) | P value | Beta (95%CI) | P value |
| Study Design |  | **0.002** |  | **<0.001** |  | **0.001** |  | **<0.001** |
| Cross-sectional | Reference |  | Reference |  | Reference |  | Reference |  |
| Prospective | -3.64 (-8.97 to 1.70) |  | -2.63 (-7.77 to 2.52) |  | -3.37 (-8.55 to 1.82) |  | -3.52 (-8.77 to 1.73) |  |
| RCT | 1.57 (-6.87 to 10.01) |  | 0.63 (-8.75 to 7.48) |  | -2.29 (-10.59 to 6.02) |  | -0.71 (-9.02 to 7.59) |  |
| Surveys | 1.99 (-6.57 to 10.56) |  | 4.40 (-3.87 to 12.66) |  | 4.45 (-3.84 to 12.74) |  | 2.63 (-5.81 to 11.07) |  |
| Retrospective chart review | -3.31 (-14.02 to 7.39) |  | -2.83 (-13.30 to 7.64) |  | -3.07 (-8.26 to 2.12) |  | -2.39 (-12.87 to 8.08) |  |
| Quasi RCT | **-37.48 (-55.09 to -19.86)** |  | **-38.55 (-55.46 to -21.65)** |  | **-37.78 (-55.04 to -20.51)** |  | **-41.22 (-58.50 to -23.94)** |  |
|  |  |  |  |  |  |  |  |  |
| WHO region |  | 0.13 |  |  |  |  |  |  |
| Africa | Reference |  |  |  |  |  |  |  |
| Americas | 3.51 (-12.78 to 19.79) |  |  |  |  |  |  |  |
| Eastern Mediterranean | 9.84 (-2.05 to 21.73) |  |  |  |  |  |  |  |
| S.E. Asia | **6.22 (0.80 to 11.65)** |  |  |  |  |  |  |  |
| Western Pacific | -8.60 (-25.20 to 8.00) |  |  |  |  |  |  |  |
| Multi- regional | -0.35 (-35.99 to 36.70) |  |  |  |  |  |  |  |
|  |  |  |  |  |  |  |  |  |
| Recruitment setting |  | 0.77 |  |  |  |  |  |  |
| Community based | Reference |  |  |  |  |  |  |  |
| Hospitals or clinics | -1.67 (-6.75 to 3.42) |  |  |  |  |  |  |  |
| Prisons or residential facility | 2.86 (-5.79 to 11.51) |  |  |  |  |  |  |  |
| Workplaces | -0.50 (-9.88 to 8.89) |  |  |  |  |  |  |  |
|  |  |  |  |  |  |  |  |  |
| Type of population screened |  | 0.88 |  |  |  |  |  |  |
| General population | Reference |  |  |  |  |  |  |  |
| Contacts | -1.68 ( -7.96 to 4.60) |  |  |  |  |  |  |  |
| PLWH | -0.56 (-6.86 to 5.75) |  |  |  |  |  |  |  |
| High risk for TB exposure^a^ | -3.83 (-11.85 to 4.18) |  |  |  |  |  |  |  |
| High risk for active TB^b^ | -2.46 (-10.52 to 5.60) |  |  |  |  |  |  |  |
|  |  |  |  |  |  |  |  |  |
| Age |  | 0.45 |  |  |  |  |  |  |
| Adults | Reference |  |  |  |  |  |  |  |
| Adults and children | -2.79 (-8.72 to 3.15) |  |  |  |  |  |  |  |
| Children only | -3.56 (-10.44 to 3.32) |  |  |  |  |  |  |  |
|  |  |  |  |  |  |  |  |  |
| Year of publication |  | 0.35 |  |  |  |  |  |  |
| 2011 to 2016 | Reference |  |  |  |  |  |  |  |
| 2000 to 2010 | -1.37 (-6.71 to 3.97) |  |  |  |  |  |  |  |
| 1980 to 1999 | -6.00 (-14.23 to 2.23) |  |  |  |  |  |  |  |
|  |  |  |  |  |  |  |  |  |
| Study quality rating |  | **0.003** |  | **<0.001** |  | **<0.001** |  | **<0.001** |
| High quality | Reference |  | Reference |  | Reference |  |  |  |
| Moderate quality | -10.55 (-18.02 to -3.09) |  | **-12.11 (-19.28 to -4.93)** |  | **-11.06 (18.46 to -3.65)** |  | **-10.86 (-18.17 to -3.54)** |  |
| Low quality | -6.84 (-11.91 to -1.77) |  | **-10.35 (-15.37 to -5.32)** |  | **-10.42 (-15.51 to -5.33)** |  | **-8.35 (-13.38 to -3.33)** |  |
|  |  |  |  |  |  |  |  |  |
| Screening modality |  | 0.53 |  |  |  |  |  |  |
| Symptom screening | Reference |  |  |  |  |  |  |  |
| CXR | 2.79 (-3.99 to 9.58) |  |  |  |  |  |  |  |
| Lab Screening^c^ | -2.96 (-8.57 to 2.64) |  |  |  |  |  |  |  |
| TST | -1.56 (-27.76 to 24.63) |  |  |  |  |  |  |  |
|  |  |  |  |  |  |  |  |  |
| Diagnostic modality |  | **0.02** |  | **0.02** |  |  |  |  |
| Microscopy | Reference |  | Reference |  |  |  |  |  |
| Culture &/or Gene Xpert | **-7.98 (-13.53 to -2.43)** |  | **-9.74 (-15.14 to -4.34)** |  |  |  |  |  |
| CXR | -5.07(-12.22 to 2.09) |  | -5.76 (-12.62 to 1.11) |  |  |  |  |  |
|  |  |  |  |  |  |  |  |  |
| Combined Screening and Diagnostic Algorithm |  | **0.02** |  |  |  | **0.005** |  |  |
| Symptom – Microscopy | Reference |  |  |  | Reference |  |  |  |
| Symptom – CXR | -5.75 (-13.37 to 1.88) |  |  |  | -6.36 (-13.80 to 1.08) |  |  |  |
| Symptom – Culture/Xpert | **-10.72 (-17.35 to-4.09)** |  |  |  | **-12.07 (-18.45 to -5.68)** |  |  |  |
| CXR – Culture/ Xpert | -1.85 (-10.02 to 6.51) |  |  |  | -4.54 (-12.70 to 3.61) |  |  |  |
| Lab^d^ – Culture/Xpert | **-9.98 (-17.10 to -2.86)** |  |  |  | **-11.35 (-18.55 to -4.16)** |  |  |  |
| Other^d^ | -7.51 (-18.15 to 3.13**)** |  |  |  | -7.11 (-17.48 to 3.25) |  |  |  |
|  |  |  |  |  |  |  |  |  |
| Diagnostic test using GeneXpert |  | 0.86 |  |  |  |  |  | 0.93 |
| GeneXpert not used | Reference |  |  |  |  |  | Reference |  |
| GeneXpert used | -0.62 (-7.88 to 6.64) |  |  |  |  |  | 0.32 (- 6.65 to 7.29) |  |

a High risk for TB exposure: health care worker, prisoner, refugee.

b High risk for active TB: diabetes mellitus, pregnancy, miners.

c Includes screening using AFB smear (94%), culture (71%), or GeneXpert (8%)

d Other = microscopy for screening and diagnosis (5), CXR for screening and diagnosis (5), CXR for screening then microscopy for diagnosis (2), TST for screening then culture for diagnosis (1), and microscopy for screening then CXR for diagnosis (2)
