# Supplementary material for: Yield and Coverage of Active Case Finding Interventions for Tuberculosis Control:A Systematic Review and Meta-analysis
Source: Tuberc Res Treat. 2022 Jun 30;2022:9947068. doi: 10.1155/2022/9947068 (PMC9274229; doi:10.1155/2022/9947068)
Supplement: Supplementary 5 — Supplemental Material 5: Table Showing Pooled Estimates of TB Yield, Based on 277 Populations with Data on Numerator and Denominator. [file 9947068.f5.docx]

**Supplemental Material 5: Pooled Estimates of TB Yield, Based on 277 Populations with Data on Numerator and Denominator**

| **Characteristics** | **Number Screened** | **New TB Cases** | **Pooled Yield**  **Estimate (95% CI)** |
| --- | --- | --- | --- |
| Overall | 25,652,139 | 55,549 | 3.15 (2.93 – 3.37) |
| Study Design  RCT  Quasi RCT  Prospective  Cross-sectional  Survey  Retrospective chart review | 453,431  534,359  804,220  22,215,797  1,612,560  31,772 | 5,405  1,770  10,196  34,563  2,514  1,101 | 4.61 (3.12 – 6.37)  0.89 (0.41 – 1.56)  4.42 (3.68 – 5.23)  2.89 (2.58 – 3.21)  0.75 (0.54 – 1.00)  8.19 (4.32 – 13.14) |
| WHO region  Africa  Southeast Asia  Eastern Mediterranean  Western Pacific  The Americas  Multi-regional | 1,643,626  23,410,981  568,766  15,373  12,686  707 | 21,213  31,436  1,720  576  519  85 | 4.44 (3.93 – 4.49)  1.31 (1.10 – 1.52)  1.12 (0.35 – 2.29)  9.03 (1.14 – 23.04)  3.17 (0.65 – 7.44)  12.02 (9.83 – 14.63) |
| Recruitment setting  Community  Hospital or clinic  Prisons or residential facility  Workplaces  Internally-displaced persons camp | 24,104,797  1,290,837  116,549  138,254  1,702 | 40,662  8,436  2,045  4,139  267 | 1.78 (1.57 – 1.99)  6.85 (6.08 – 7.66)  2.00 (0.92 – 3.44)  2.63 (2.03 – 3.32)  15.62 (13.93 – 17.39) |
| Type of population screened  Contacts  PLWH  General population  High risk for TB exposure^a^  High risk for active TB^b^ | 724,854  81,400  24,528,756  150,493  166,636 | 11,513  5,406  31,877  2,203  4,550 | 3.34 (2.85 – 3.86)  9.05 (7.02 – 11.30)  1.23 (1.08 – 1.39)  1.57 (0.87 – 2.45)  3.63 (2.62 – 4.79) |
| Age  Children only  Adults only  Adults and children | 145,512  22,089,548  3,417,079 | 2,243  33,502  19,804 | 6.32 (4.43 – 8.50)  2.92 (2.64 – 3.22)  2.74 (2.29 – 3.22) |
| Year of Publication  2011 to 2016  2000 to 2010  1980 to 1999 | 23,804,717  702,731  1,144,691 | 47,715  5,677  2,157 | 3.22 (2.93 – 3.52)  3.98 (3.28 – 4.73)  0.89 (0.67 – 1.13) |
| Study quality rating  High quality  Moderate quality  Low quality | 3,259,724  367,847  22,024,568 | 30,832  3,728  20,989 | 3.96 (3.58 – 4.36)  2.57 (1.83 – 3.43)  1.86 (1.64 – 2.09) |
| Screening modality  Symptom screening  Lab screening^c^  CXR  TST | 24,832,174  224,133  595,514  318 | 40,084  5,610  9,842  13 | 2.22 (2.03 – 2.42)  6.57 (5.29 – 7.98)  3.33 (2.69 – 4.05)  4.09 (2.40 – 6.87) |
| Diagnostic modality  Culture &/or GeneXpert MTB/RIF  Microscopy  CXR | 3,016,102  20,865,019  1,771,018 | 23,437  23,724  8,388 | 3.69 (3.34 – 4.06)  2.00 (1.58 – 2.46)  3.55 (2.95 – 4.20) |
| Combined Screening and Diagnostic Algorithm  Symptom – Microscopy  Symptom – CXR  Symptom – Culture/Xpert  CXR – Culture/Xpert  Lab^c^ – Culture/Xpert  Other^d^ | 20,826,814  1,724,523  2,280,837  541,016  193,931  85,018 | 23,058  7,585  9,441  8,862  5,121  1,482 | 1.93 (1.50 – 2.41)  3.31 (2.71 – 3.98)  2.15 (1.86 – 2.45)  3.39 (2.66 – 4.20)  6.94 (5.50 – 8.53)  3.71 (2.39 – 5.29) |
| Diagnosis using GeneXpert MTB/RIF  GeneXpert MTB/RIF not used  GeneXpert MTB/RIF used | 24,554,096  1,098,043 | 49,240  6,309 | 3.02 (2.79 – 3.27)  4.11 (3.43 – 4.83) |

CI = confidence interval, CXR = chest x-ray, PLWH = people living with HIV infection, RCT = randomized controlled trial, TST = tuberculin skin test

^a^ High risk for TB exposure: health care worker, prisoner, refugee.

^b^ High risk for active TB: diabetes mellitus, pregnancy, miners.

^c^ Includes initial screening using AFB smear (94%), culture (71%), or GeneXpert MTB/RIF (8%)

^d^ Other = microscopy for screening and diagnosis (5), CXR for screening and diagnosis (5), CXR for screening then microscopy for diagnosis (2), TST for screening then culture for diagnosis (1), and microscopy for screening then CXR for diagnosis (2)
